# In vitro approaches to assess respiratory toxicity from volatile organic compounds: an in-depth review

**DOI:** 10.1093/toxsci/kfag110

**Published:** 2026-08-20

**Authors:** Olivia C G Lampe, Eva C M Vitucci, Carolyn L Cannon, Kyle Holland, Margaret J Foster, Natalie M Johnson

**Affiliations:** Department of Environmental and Occupational Health, Interdisciplinary Program in Toxicology, Texas A&M University, College Station, TX 77843, United States; Department of Environmental and Occupational Health, Interdisciplinary Program in Toxicology, Texas A&M University, College Station, TX 77843, United States; Department of Microbial Pathogenesis and Immunology, Texas A&M University, College Station, TX 77843, United States; Medical Sciences Library, Texas A&M University, College Station, TX 77843, United States; Medical Sciences Library, Texas A&M University, College Station, TX 77843, United States; Department of Environmental and Occupational Health, Interdisciplinary Program in Toxicology, Texas A&M University, College Station, TX 77843, United States

**Keywords:** human lung epithelial cells, air–liquid interface culture, submerged cell culture, dose characterization, oxidative stress

## Abstract

Volatile organic compounds (VOCs) are ubiquitous inhaled pollutants. This review showcases current literature utilizing in vitro models of the human respiratory system to characterize the toxicity of VOCs. To map the existing evidence base, we conducted a scoping review following systematic search and screening procedures. Comprehensive searches of MEDLINE, Embase, Web of Science, CINAHL, PubMed, and CENTRAL identified 3,052 records. After screening, 144 original studies evaluating VOC exposures in human lung epithelial models met inclusion criteria. Overall, the current literature reflects substantial heterogeneity in cell models, exposure systems, and endpoints. Among 105 unique VOCs evaluated, acrolein, formaldehyde, toluene diisocyanate, and benzene were most frequently studied. Most investigations used submerged culture systems with liquid-phase VOC application, whereas fewer employed air–liquid interface (ALI) exposures that better mimic inhalation. Cytotoxicity, oxidative stress, proinflammatory signaling, and apoptosis were the most commonly measured endpoints, with oxidative stress frequently identified as an upstream driver of inflammatory and cytotoxic responses. However, mechanistic depth varied, and studies examining metabolism, barrier function, morphology, or transcriptomic regulation were relatively uncommon. Notably, chronic or repeated exposures were rarely conducted. Overall, this review highlights the variety of VOC exposure methods and commonly assessed biological endpoints, as well as the critical lack of more detailed mechanistic studies and somewhat limited VOC/mixture evaluation. The field is expansive but methodologically fragmented, underscoring the need for broader use of human respiratory cell models, more physiologically relevant exposure systems, improved dose characterization, and greater mechanistic resolution to advance understanding and throughput for VOC-induced pulmonary toxicity studies.

## Introduction

Volatile organic compounds (VOCs) are ubiquitous environmental pollutants present in indoor and outdoor air, arising from sources such as industrial emissions, vehicular exhaust, household products, and building materials (David and Niculescu 2021). Exposure to VOCs has been associated with a range of adverse respiratory outcomes, including airway inflammation, impaired lung function, and exacerbation of chronic respiratory diseases (Kwon et al. 2018; Lv et al. 2023; Chen et al. 2025). Despite their widespread presence, the pulmonary toxicity of VOCs is less characterized than that of other contaminants designated “criteria” air pollutants by the US Environmental Protection Agency (EPA), including particulate matter and ozone (Aufderheide et al. 2003; Upadhyay et al. 2022). Moreover, the underlying cellular mechanisms of toxicity remain poorly understood.

In vitro models of the human respiratory epithelium offer a controlled platform to investigate VOC-induced toxicity and elucidate mechanistic pathways (Galezowska et al. 2016). These models include immortalized cell lines, such as A549 and BEAS-2B, as well as primary human bronchial and small airway epithelial cells. Cells can be cultured under submerged conditions or at the air–liquid interface (ALI), with ALI cultures providing a more physiologically relevant representation of inhalation exposures. In vitro systems allow for precise control of exposure conditions; however, they have inherent limitations, including challenges in dose delivery, frequent absence of multicellular interactions, and differences from in vivo lung physiology (Cidem et al. 2020).

Prior in vitro investigations of VOC respiratory toxicity have predominantly focused on a limited number of compounds and variety of models (Galezowska et al. 2016). Given the heterogeneity of methods and incomplete understanding of VOC-induced effects, a systematic synthesis of the literature is needed. A scoping review is particularly suited to map the existing evidence, identify methodological approaches, highlight commonly assessed endpoints, and pinpoint critical gaps in research.

The objective of this review was to map and synthesize the literature on in vitro studies of the response of human respiratory epithelial cells to VOC exposure. The review focuses on exposure methods, biological endpoints, mechanistic insights, and gaps in chronic and physiologically relevant exposures. By providing a comprehensive overview, this review aims to inform future experimental designs and advance mechanistic understanding of VOC-induced pulmonary toxicity.

## Review of the literature

### Study question

The objective of this review was to highlight the variety of in vitro approaches used to investigate VOC human respiratory exposures and identify critical gaps in the literature discussing VOC-induced effects in human epithelial cell-based models.

#### Data sources

**Fig. 1.**
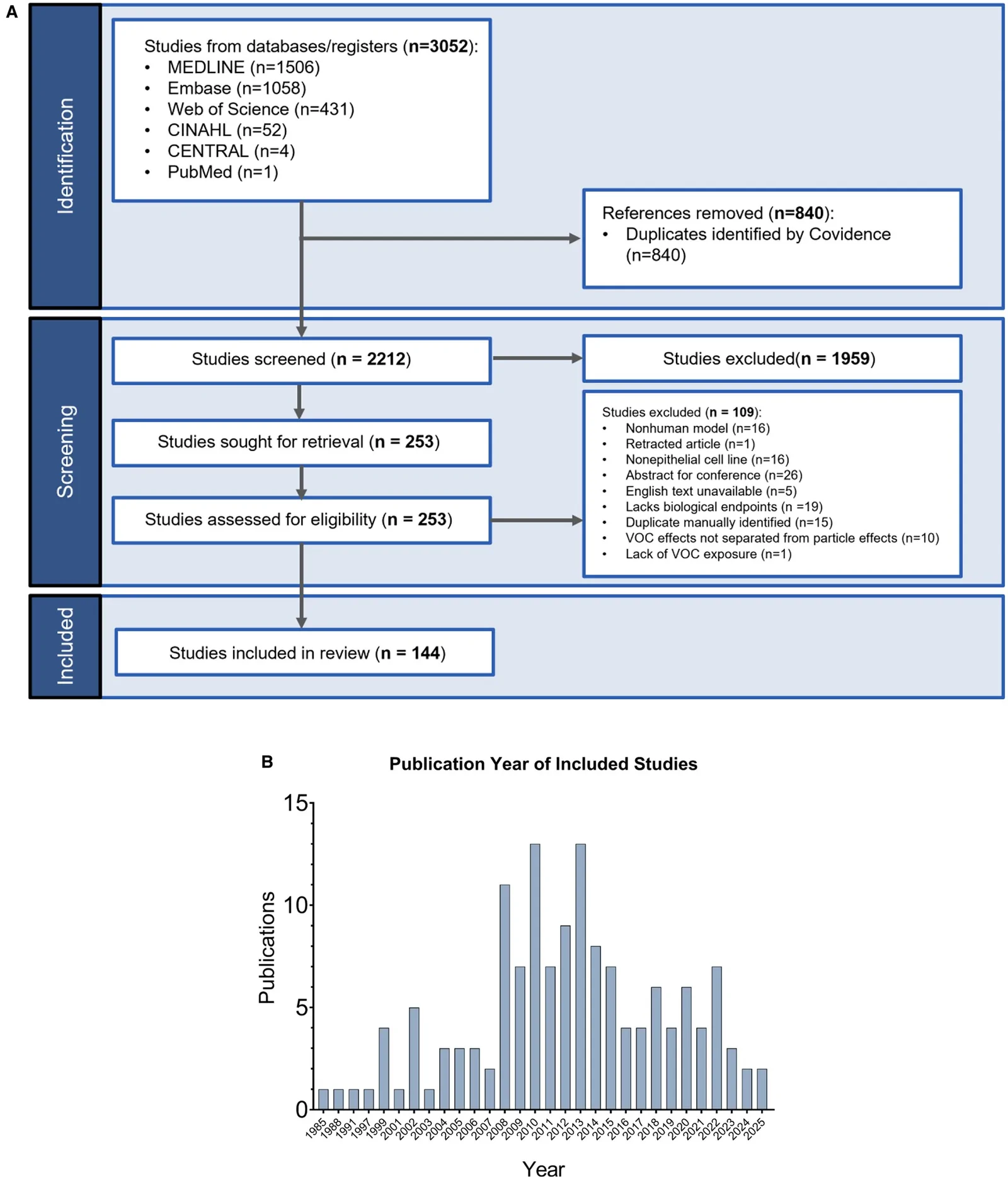
(A) Flowchart showing the literature search and screening process for studies relevant to in vitro VOC human respiratory exposures. (B) Timeline of the publication year of the included studies in this review.

In conjunction with a librarian and an information specialist who focus on evidence synthesis and systematic searching, the team developed a search strategy that combined 3 concepts: Lungs and related anatomy, cell models or cultures, and volatile organic compounds. For each concept, a list of known and potential synonyms was combined with appropriate thesaurus terms for each of the 6 databases searched: MEDLINE (Ovid), Embase (Ovid), Web of Science Core Collection (Web of Science), CINAHL, PubMed, and CENTRAL (Cochrane Library). The complete search terms can be found in Table S1. Searches were conducted on April 16, 2025 with no database limits imposed. Articles were retrieved and uploaded to Covidence for deduplication and screening. A total of 3,052 studies were identified from the databases MEDLINE, Embase, Web of Science, CINAHL, PubMed, and CENTRAL. Eight hundred forty duplicates were identified by Covidence, and the remaining studies were manually screened by 2 of the authors. This initial screening resulted in 253 studies advancing to full-text review. An additional 109 studies were excluded if: It was a duplicate, it lacked biological endpoints, it was a conference abstract, English text was unavailable, nonhuman or nonepithelial cell lines were used, or VOC toxicity data could not be separated from particulate matter effects. In summary, 144 studies were selected to be included in this scoping review (Fig. 1; Table S2).

#### Study selection

Original studies that evaluated VOC exposure using in vitro human respiratory models were selected. Two reviewers independently screened titles and abstracts in Covidence to determine eligibility. Discrepancies between eligibility were resolved by a third reviewer. Studies were excluded if they did not include a human lung epithelial cell line, the study was an abstract for a conference, VOC effects could not be separated from particulate matter effects, English text was unavailable, the article was retracted, cells were not exposed to a VOC, or the study did not measure any biological endpoints. Studies that investigated exhaled VOCs as markers for respiratory diseases were also excluded. The same criteria were utilized during the full-text review, where 2 reviewers independently reviewed each study.

#### Data extraction

Data extraction was independently completed by 2 reviewers. Data extracted by each reviewer was independently reviewed by a third reviewer to maintain accuracy and resolve any discrepancies. Data extraction included the characteristics of the study populations, including the investigated cell type, investigated VOC, cell culture exposure method, exposure medium, dosage, time points, and study results. The VOC application methods are described as vapor, diffusion, liquid, and hanging drop. Vapor is defined as a gaseous VOC application to the cell culture. Diffusion denotes that either an applied gaseous VOC must diffuse through a liquid medium to reach the cell culture or that a VOC applied in a liquid form must evaporate to reach the cell culture. Liquid is defined as a direct liquid application of VOCs to the cell culture. Hanging drop involves a drop of culture media containing cells hung from the top of a flask, whereas a liquid VOC is injected into the flask. All VOC doses were converted into ppm for ease of comparison by the reviewers.

### The A549 cell line dominates the in vitro investigation of VOC exposure effects

The epithelium of the human respiratory system is a ciliated pseudostratified epithelium composed of multiple cell types. The alveolar region of the lungs is the terminal end of the system at which gas exchange occurs, and the bronchial region is the middle passageway that connects the alveoli to the upper respiratory zone (Chaudhry et al. 2024). There was a total of 13 different cell lines used within the studies included in this review that aim to reflect these different regions of the lung (Table S3).

#### Alveolar

**Fig. 2.**
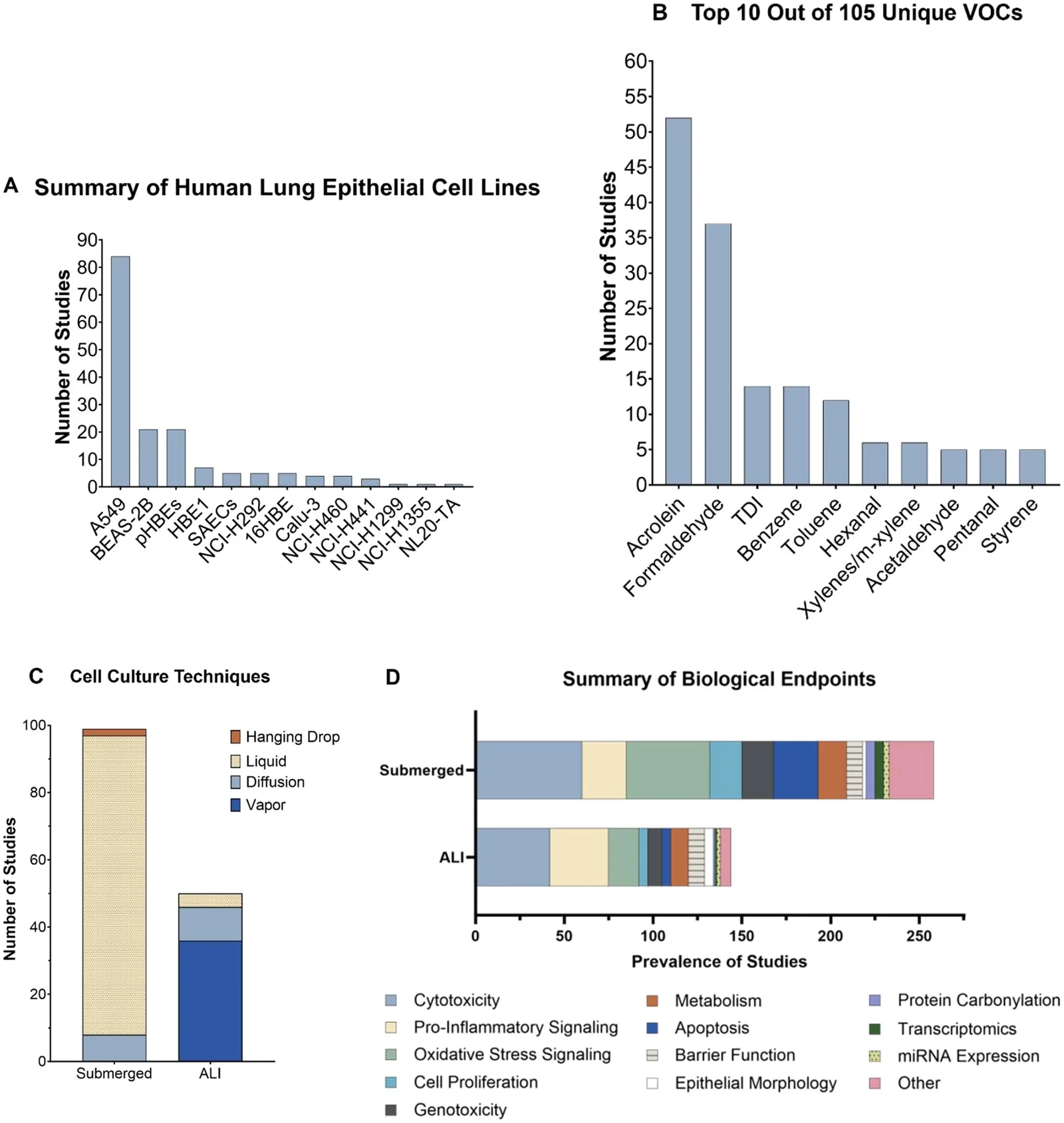
(A) Distribution of human lung epithelial cell lines observed in this review. Studies (*n* = 22) that utilized more than one cell line are denoted by an asterisk (*) in Table S2. (B) Distribution of VOCs investigated in the in vitro human lung studies. (C) Breakdown of VOC application methods by cell culture method. Hanging drop was considered an application method separate from diffusion because of its unique methodology in which a “drop” of cells suspended in a liquid medium were exposed to a liquid VOC that had to evaporate before diffusing through the drop of cells. This approach can also be classified as a double diffusion method, i.e. liquid to gas to liquid. (D) Summary of the commonly investigated biological endpoints separated by cell culture method.

The overwhelming majority of studies (*n* = 84), accounting for approximately half of all investigations in this review, utilized the A549 cell line which was isolated from a pulmonary adenocarcinoma extracted from a Caucasian 58-yr-old male in 1972 (Lieber et al. 1976) (Fig. 2A). These squamous cells are relatively easy to cultivate as a monolayer hence their popularity. The A549 cell line has been characterized as representative of alveolar type II pneumocytes of the human lung due to the presence of multilamellar cytoplasmic inclusion bodies, and as such have been used extensively in cancer research and for modeling respiratory function (Lieber et al. 1976). Alveolar type II pneumocytes have the primary function of transepithelial movement of water and producing and secreting pulmonary surfactant which keeps the alveoli from collapsing. They communicate with type I pneumocytes which are responsible for facilitating gas exchange (Brandt and Mandiga 2023). Notably, under submerged cell culture conditions, A549 cells do not secrete surfactant and lack functional tight junctions, but when brought to ALI, they increase their expression of markers of tight junctions including ZO-1 and occludin, and form functional tight junctions (Petpiroon et al. 2023). The A549 and the NCI-H441 cell lines (another human alveolar epithelial cell line) were the only cells used to investigate exposure effects in the human alveolar epithelium. Notably, fewer than 10 studies used the NCI-H441 in this review despite their ability to overcome the limitations of the A549 cell line and express both surfactant and functional tight junction proteins under submersion (Salomon et al. 2014; Vitucci et al. 2024). All other cell lines have been characterized as representative of bronchial or unspecified lung epithelium.

#### Bronchial

The remainder of studies evaluated in this review, approximately 60 studies, investigated VOC exposure effects using a range of different bronchial epithelial cell models, with 2 of the 11 immortalized cell lines created from female donors and the remainder sourced from male donors (Table S3). The BEAS-2B cell line, an immortalized cell line derived from normal, bronchial epithelial cells, was the second-most utilized cell line in this review and used in 13% of studies. This cell line was established as a nontumorigenic epithelial cell line immortalized through an adenovirus 12-SV40 transfection of “normal” human bronchial epithelial cells (Han et al. 2020; Lee and Ryu 2023). Additionally, the BEAS-2B cell line has features associated with mesenchymal stem cells (MSCs) such as surface markers and adipogenic differentiation potential (Han et al. 2020). Furthermore, the BEAS-2B cell line may present as morphologically atypical cells that possess multiple lobes and nuclei which deserves consideration when exposing this cell line to carcinogens (Lee and Ryu 2023).

All other cells lines, HBE1, NCI-H292, 16HBE, Calu-3, NCI-H460, NCI-HI299 (*n* = 1), NCI-H1355 (*n* = 1), and NL20-TA (*n* = 1), were individually used in less than 5% of studies and were derived from either normal tissue or carcinomas. Of this list, the 16HBE and NL20-TA lines were created from normal tissue. A commonly utilized cell line, the 16HBE cell line was isolated from a 1-yr-old male patient and immortalized with the origin-of-replication defective SV40 plasmid (pSVori-) (Cozens et al. 1994). 16HBEs share characteristics of normal human bronchial epithelial cells and are commonly used to model airway barrier function. The cells have a cobblestone morphology, form tight junctions, and have the ability to form directional ion-transport. In comparison to other human respiratory cell lines, 16HBEs express high levels of the protein cystic fibrosis transmembrane conductance regulator (CFTR) (Cozens et al. 1994). CFTR controls the flow of chloride ions in any organ that produces mucus, and dysfunction of CFTR in the lungs results in the mucus lining becoming thicker which increases the risk of bacterial infections and asthma complications (Koumpagioti et al. 2023). As such, the 16HBE cell line has been used to model asthma, COPD, cystic fibrosis, drug transport, and, to a lesser extent, lung cancer in prior toxicological studies. When brought to ALI, 16HBEs do not differentiate into the main bronchial phenotypes: Goblet, basal, and ciliated cells. The differentiated 16HBEs are polarized, maintain tight junctions, form pseudo-cilia, but lack goblet and basal cells (Van den Bossche et al. 2023).

The strength of the 16HBE cell line in its ability to form tight junctions and directional ion-transport. As such, 16HBEs can model changes to barrier function, which is important because many respiratory diseases are exacerbated by a leaky epithelium. However, the high expression of CFTR can make TEER measurements, a popular parameter for barrier function, complicated to interpret. In one study, Inhibition of CFTR resulted in increased TEER values when compared with 14C-d-mannitol flux rates which held stable under similar stimuli (Callaghan et al. 2020). In the same study, TEER proved to be more variable, and to spontaneously and significantly decrease after initial barrier formation, whereas 14C-d-mannitol flux rates did not significantly change.

#### Adenocarcinomas

Seven out of 11 of the immortalized cell lines included in this review were derived from carcinomas, with the HBE1 cell line developed from an individual with pulmonary fibrosis. Calu-3, NCI-H460, and NCI-H292 are 3 cell lines derived from cancer cells that are considered representative of the human bronchial epithelium. Calu-3 is judged as relatively easy to culture and expresses high levels of the tight junction proteins ZO-1 and occludin at both ALI and in submerged cultures (Petpiroon et al. 2023). NCI-H460 is an adherent nonsmall lung cancer cell line derived from metastatic pleural fluid. These cells have been used in drug research as well as in vitro models for cellular and molecular mechanisms involved in tumor development and metastasis (Zhou et al. 2020; Li et al. 2023). NCI-H292 is another adherent nonsmall lung cancer cell line that was derived from a lymph node metastasis. This cell line retains mucoepidermoid characteristics in culture (Carney et al. 1985).

Monocultures of cancer cell lineages have significantly different gene expression than cell lines derived from normal cells and are often cultured with immune cell types to replicate physiological functions of the human lung because most apoptosis-signaling requires an immune component (Petpiroon et al. 2023). As with all studies of single cell lines, conclusions pertaining to overall lung health are limited given that the lung is a complex, spatially diverse organ with over 40 different cell types (Franks et al. 2008; Khan and Carey 2025).

#### Population variability

Primary cells offer potential evaluation of inter-individual variability and focus on susceptible populations, such as pediatrics. Sixteen studies used primary human bronchial epithelial (pHBEs or SAEC) cells in their cell culture model. Ten of those studies exposed primary cells at ALI, 5 used a submerged model, and one study exposed primary cells using both an ALI and a submerged model (Franks et al. 2008). The study that compared VOC-induced effects between ALI and submerged models found that submerged primary cells had dampened responses compared with primary cells exposed at ALI (Dwivedi et al. 2018). Generally, studies used 1 to 3 adult donors, but 6 studies did not report donor information. Overwhelmingly, studies utilizing primary cells cultured and exposed the cells at ALI to either acrolein, formaldehyde, or TDI. Interestingly, despite the higher potential for primary cells to mimic xenobiotic metabolism compared with immortalized cell lines, almost none of the studies utilizing primary cells explored metabolism (Pan et al. 2009). Also, structural changes were not heavily investigated; 2 papers investigated how TDI and formaldehyde, respectively, affected ciliary function (Lange et al. 1999; Le et al. 2025). TDI was observed binding to the cilia of bronchial epithelial cells leading to impaired mucociliary activity, whereas formaldehyde decreased ciliary beating. In summation, the 2 main endpoints investigated in VOC-exposed primary cell studies were proinflammatory signaling and barrier function. As such, the traditional advantages of using primary cells were not fully captured in the VOC literature, which includes changes to cellular structures (i.e. cilia, mucous), metabolism, and population variability.

### Heterogeneity and validation of VOCs across in vitro epithelial exposure studies

This review identified a wide range of VOCs that have been investigated for respiratory toxicity; however, the investigation was not evenly distributed among all VOCs. Reasonably, most studies investigated the common VOCs, acrolein, and formaldehyde. These VOCs are present in cigarette smoke, ambient outdoor emissions, and in certain occupational spaces (Sinharoy et al. 2019). However, there are numerous additional VOCs with likely equivalent prevalence that are lacking robust investigation of their respiratory exposure effects (David and Niculescu 2021). Moreover, another gap identified through this review was the lack of validation of the delivered VOC doses.

#### Scope of investigated VOCs

There was a total of 105 unique VOCs that were evaluated in this review (Fig. 2B). Of these 105 VOCs, the effects of acrolein on the human lung epithelium were investigated in 52 separate studies. Formaldehyde was investigated in 38 separate studies, and toluene diisocyanate (TDI) and benzene were each investigated in 14 studies, respectively. As such, the effects of these 4 VOCs make up the majority of current in vitro literature investigating VOC effects on the human lung epithelium. Fourteen studies investigated mixture effects. The full list of mixtures can be found in Table S4.

#### Dose validation of investigated VOCs

One of the main challenges of in vitro VOC exposures is dose uniformity within and between studies. The majority of studies (*n* = 110) did not report any validation for delivered dose irrespective of the exposure system. For all systems, supplementary analytical equipment must be used to confirm delivered dose. Of the studies that did report delivered dose (*n* = 34), the 2 main analytical methods were gas chromatography-mass spectrometry (GC-MS) and gas chromatography-flame ionization detection (GC-FID). A breakdown of the dose validation techniques can be found in Fig. S1.

### Cell culture exposure methods utilized in the in vitro epithelial exposure studies

Submerged cultures, defined as cells cultured under a layer of liquid cell culture media, are the traditional method utilized in studies investigating exposure effects to chemicals or toxicants of interests. Using this approach, a toxic agent is dissolved or suspended in cell culture media and applied directly to the cells (Lenz et al. 2013). However, growing discussion around the relevancy of submerged exposure methods for the investigation of inhalation exposure effects has led to the development of the air–liquid interface (ALI) culture method and exposure system (Lacroix et al. 2018; Allouche et al. 2025). Using this approach, cells are typically cultured on a substrate such as a Transwell insert, with their apical surface exposed to air and their basolateral surface exposed to cell culture media. Once at ALI, cells are then either exposed to the chemical of interest dissolved in a liquid or directly exposed to a chemical of interest in gaseous form. Both cell culture methods have their advantages and disadvantages, and both have been utilized to investigate the in vitro exposure effects of VOCs (Kaur et al. 2022).

#### Submerged exposure methods

A majority of studies (*n* = 99) utilized a submerged exposure method in which lung cells are completely submerged in culture media conditions during the exposure (Fig. 2C). Of these studies, 89 applied the VOC as a liquid and 8 studies exposed submerged cells to a gaseous VOC that must diffuse through the liquid medium to reach the cell culture. Lastly, 2 submerged studies utilized a specific form of diffusion, denoted as the “hanging drop,” in which cells are suspended in a hanging droplet of media from the lid of an exposure chamber and VOCs, introduced to the exposure chamber via liquid application, must evaporate and diffuse again through the cell culture medium to reach the cells. Another way of thinking about hanging drop would be “double diffusion” (Chen et al. 2002; Bakand et al. 2006).

The submerged application is the simplest cell culture model available but has 2 major limitations: Difficulty in deriving the delivered dose and lack of physiological relevance (Lenz et al. 2013). The volatility and hydrophilicity of the VOC, as well as the depth of the culture media on top of the cells, can affect delivered dose (Teeguarden et al. 2007; National Research Council 2009). For diffusion especially, additional analytical methods, such as gas chromatography-mass spectrometry (GC-MS), are needed to estimate delivered dose (Chen et al. 2002). A more relevant VOC delivery method that better models in vivo inhalation exposure involves the direct contact of a vapor with the lung epithelium. Many of the newer studies in this review used ALI cell culture models rather than submerged models (Anderson et al. 2013).

#### ALI exposure methods

The minority of in vitro studies (*n* = 50) investigating how VOCs affect human respiratory health utilized an ALI cell culture model (Fig. 2C). The ALI exposure system improves upon the physiological relevance of the submerged cell culture model by exposing cells, usually grown on Transwell inserts, to air on their apical surface. This system emulates the environment of respiratory epithelial cells in vivo that would encounter vapors and aerosols diffusing through only the microns-thick airway surface liquid during an inhalation exposure. This system also avoids physicochemical changes the airborne compounds may undergo when they reach the cell medium, such as chemical reactivity and particle agglomeration (Ahmad et al. 2014; Silva et al. 2023). Some challenges with the ALI cell culture model include requiring complex exposure set-up, dose delivery of aerosols and vapors requires specific equipment, and ALI cultures often require more maintenance due to frequent media changes and possible mucus accumulation (Baldassi et al. 2021; Silva et al. 2023). Of the studies using the ALI cell culture method, 36 utilized a direct gaseous VOC application, 4 utilized a direct liquid VOC application, and 10 used diffusion, where VOCs in a liquid solution placed adjacent to the ALI culture must evaporate to reach the cells.

### Introducing the variety in VOC in vitro exposure systems

**Fig. 3.**
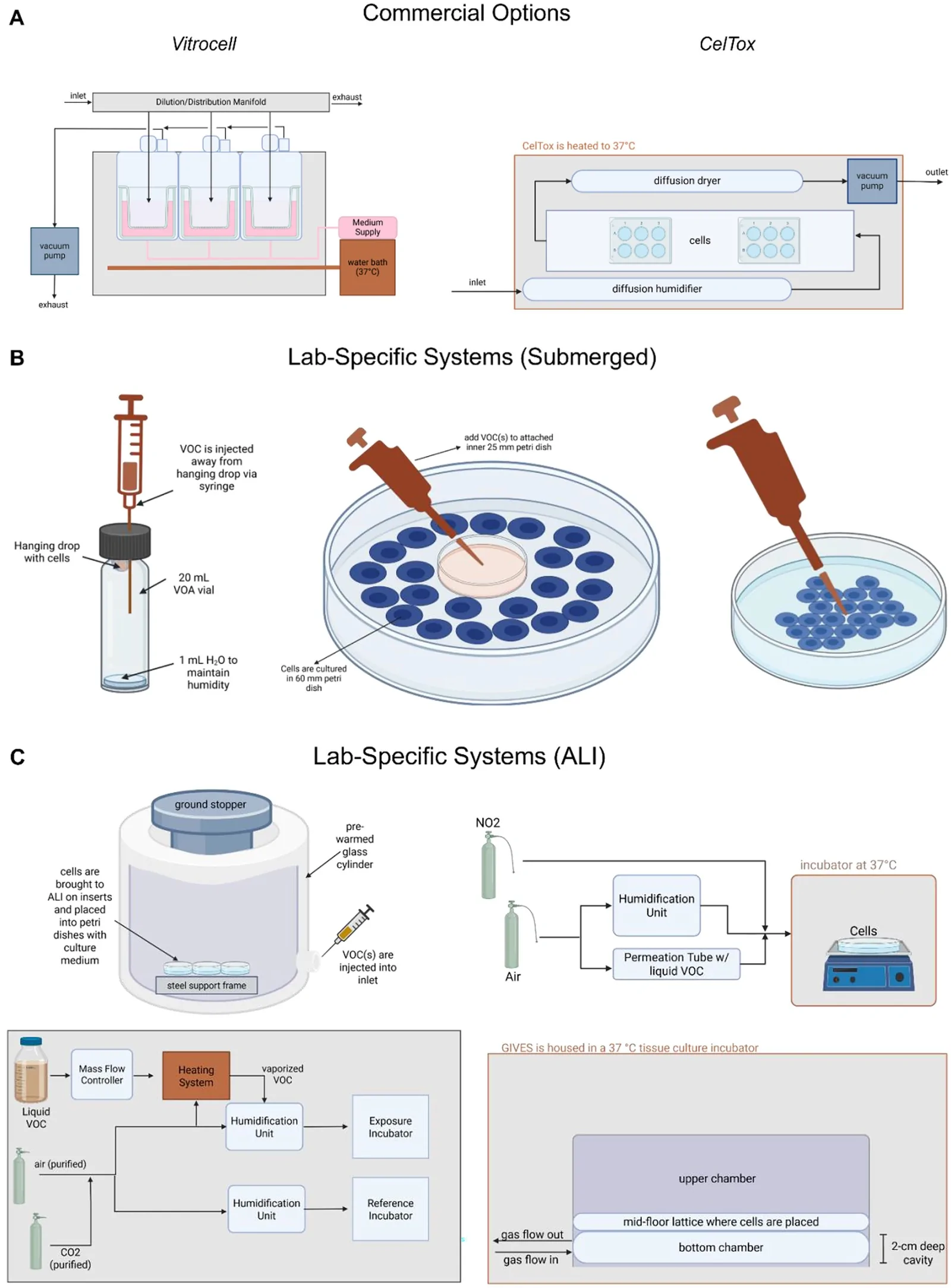
(A) Representative illustration of examples of commercial exposure systems utilized within this review. (B) Representative illustration of examples of lab-specific submerged VOC exposure systems utilized in studies included in this review. From left to right, illustrated are examples of hanging drop, diffusion, and direct liquid application methods. (C) Representative illustration of examples of lab-specific ALI VOC exposure systems utilized in studies included in this review (Chen et al. 2002; Kastner et al. 2013; Liu et al. 2013; Baldridge et al. 2015; Gostner et al. 2016).

The VOC application method was heavily dependent on the utilized exposure system. There were 2 commercial options that were found in the review: The VITROCELL and the CelTox Sampler exposure systems (Fig. 3A) (Ritter et al. 2001; Zavala et al. 2014, 2017). Both systems have been specifically designed to expose mammalian cells at ALI or tissue to vapors or aerosols. However, most studies included in this review used a custom exposure system. Examples of these custom systems are shown in Fig. 3B and C. The custom systems ranged from simple to complex as factors such as humidity, temperature, and CO_2_ were accounted for and controlled.

#### Exposure systems

The 2 commercial systems highlighted in this review were the VITROCELL and CelTox Sampler. Both are temperature and humidity controlled and are compatible with multiple types of Transwell inserts. The VITROCELL has the benefit of being highly customizable. In addition to humidity and temperature controls, the setup includes a heated aerosol inlet, a water refill system for the humidification unit, and additional heaters and ventilation fans for uniform temperature distribution throughout the system (Zimmermann et al. 2025). The CelTox Sampler is more compact than the VITROCELL. The major difference between the 2 setups is that the CelTox Sampler is limited to a single dose because the exposure chamber is not divided into subcompartments (VITROCELL 1999; Zavala 2022). Therefore, the VITROCELL system has a higher throughput when screening compounds at the air–liquid interface. However, with all ALI systems, deposition of the test compound can be affected by relative humidity, temperature of the chamber and flow stream, and the distance from the air inlet to the cells (Silva et al. 2023). This diversity poses a challenge when comparing results between labs and imposes a major problem for reproducibility, warranting more detailed methods than currently reported in the majority of publications.

### Biological endpoints investigated across in vitro VOC exposure studies

Biological endpoints were classified into 13 categories: Cytotoxicity, proinflammatory signaling, oxidative stress signaling, cell proliferation, genotoxicity, metabolism, apoptosis, barrier function, epithelial morphology, protein carbonylation, transcriptomics, miRNA expression, and other (Fig. 2D). A few categories included a broad range of related endpoints: Metabolism included mitochondrial function, glycolysis, and cytochrome P450 function. Epithelial morphology included mucin protein expression, ciliary beating, and tissue thickness. The “Other” category included unique endpoints including metalloproteinase expression, ferritin signaling, histone modifications, endoplasmic reticulum stress marker expression, keratin signaling, lipid signaling, and autophagosomal pathways.

For both exposure models, ALI and submerged, the most investigated endpoints were cytotoxicity, proinflammatory signaling, and oxidative stress signaling (Fig. 2D). In submerged models, apoptosis was equally as investigated as proinflammatory signaling (*n* = 25). The least commonly investigated endpoint for submerged models was epithelial morphology, whereas the least commonly investigated endpoints for ALI studies were protein carbonylation and transcriptomics. The majority of these studies were descriptive in nature with the minority of studies exploring deeper mechanistic insights.

#### Proinflammatory signaling

VOC exposure has been associated with pulmonary diseases and related symptoms resulting from chronic inflammation of the lung epithelium (Alford and Kumar 2021; Ogbodo et al. 2022). The majority of studies evaluated the overexpression of proinflammatory markers such as IL-8, IL-6, and monocyte chemoattractant protein-1 (MCP-1). These chemokines recruit monocytes and/or neutrophils to induce chemotaxis and are highly involved with inflammation (Deshmane et al. 2009). It was evident that there was a proinflammatory component to VOC-induced health effects with single acrolein, methacrolein, formaldehyde, chlorobenzene, benzene, toluene, p-tolualdehyde, methyl glyoxal, glyoxal, diacetyl, glutaraldehyde, 4-oxopentanal, *m*-xylene, and styrene exposures all inducing upregulation of IL-8 (Table S2). Additionally, 2 studies showed that repeated exposure to formaldehyde and toluene, respectively, increased IL-8 expression (Kastner et al. 2013; Méausoone et al. 2019).

#### Reactive oxygen species

The increase in proinflammatory signaling observed following VOC exposure appears to result from reactive oxygen species (ROS)-induced cellular dysfunction (Rahman and Adcock 2006; Zuo and Wijegunawardana 2021). ROS can disrupt cell signaling processes when intracellular ROS levels overwhelm redox modulation and can lead to the induction of oxidative stress (Martin and Barrett 2002). Multiple studies found that VOCs increased intracellular ROS levels across multiple lung epithelial cell lines within 24 h or less of exposure (Chen et al. 2002; Feltens et al. 2010; Baldridge et al. 2015). Moreover, increased intracellular ROS levels co-occurred with upregulated cell-death signaling pathways following chlorinated benzenes, trichloroethylene (TCE), and perchloroethylene (PERC) exposure, and co-occurred with induced DNA damage following low-level benzene exposure, supporting ROS generation as a primary mechanism of VOC-induced toxicity (Mörbt et al. 2011; Mascelloni et al. 2015).

#### Genotoxicity

The genotoxic effects of TDI and formaldehyde were heavily investigated in multiple papers. A few additional VOCs were also identified to have potentially genotoxic effects: Acrolein, glutaraldehyde, benzene, toluene, chlorobenzene, dibutyl phthalate, and safrole oxide. Notably, at approximately 6 ppm formaldehyde induced single-strand breaks and DNA–protein crosslinks in submerged primary normal human bronchial epithelial cells (Saladino et al. 1985). Another submerged study saw DNA strand breaks in A549 cells exposed to 3 ppm of formaldehyde (Vock et al. 1999). In contrast, using an ALI cell culture model, nonsignificant downregulation of DNA repair enzymes and nonsignificant increases in DNA damage were observed in primary cells exposed to 30 ppm formaldehyde (Le et al. 2025). This example highlights how differences in the cell culture model can affect the observed outcomes.

#### Apoptosis

Multiple VOCs are linked to the induction of apoptosis in human lung epithelial cells, including acrolein, formaldehyde, cinnamaldehyde, safrole oxide, TDI, BTEX, TCE, PERC, and chlorobenzene (Table S2). However, only acrolein- and formaldehyde-induced apoptotic pathways have been probed in detail. Acrolein appears to induce apoptosis through the intrinsic pathway (Nardini et al. 2002; Thompson and Burcham 2008; Jiang et al. 2020). The intrinsic apoptotic pathway is initiated with the activation of the central regulator of the pathway, the Bax gene, which downregulates the anti-apoptotic Bcl-2 gene resulting in programmed cell death after the downstream cleavage of BID mediated through caspase-8 (Kiraz et al. 2016). In one study, the addition of an acrolein-scavenger, γ-aminobutyric acid (GABA), to human bronchial epithelial cells exposed to 0.56 to 2.8 ppm acrolein, inhibited poly (ADP-ribose) polymerase (PARP) expression, decreased expression of caspase-8, and returned the ratio of Bax/Bcl-2 to levels similar to that of the vehicle control. Consequently, the addition of GABA significantly reduced the number of apoptotic cells. In a similar vein, formaldehyde appears to induce apoptosis through a primarily oxidative stress-mediated intrinsic pathway leading to increased Bax expression, decreased Bcl-2 expression, and induction of caspase-3 (Kastner et al. 2011; Lim et al. 2013; Jiang et al. 2020).

#### Metabolism

The metabolism endpoint included a wide range of metabolic processes, including Phase I and Phase II gene expression, mitochondrial function, and glycolysis. Gene expression differs between cell lines; one example is the difference between cytochrome P450 expression in A549 and BEAS-2B cells. Much of the toxicity of benzene is attributed to the toxicity of its metabolites: Phenols, catechols, and hydroquinones (Yardley-Jones et al. 1991). In rodents, it has been shown that CYP2E1 and CYP2F2 are the predominant enzymes that metabolize benzene in the lung and liver (Sheets and Carlson 2004). As such, the relative expression of these enzymes results in the varying ability of BEAS-2B cells to metabolize benzene compared with A549 cells and alters the resulting toxicity of benzene (Sheets et al. 2004). In A549 cells treated with acrolein, phase II genes are activated, such as NADPH quinone oxidoreductase (NQO1) and γ-glutamylcysteine synthetase (γ-GCS) suggesting Nrf2-mediated pathways (Tirumalai et al. 2002).

#### Barrier function

When exposed to formaldehyde or acrolein, both the EpiAirway cell culture model and the Calu-3 cell culture line showed decreases in barrier integrity via transepithelial electrical resistance values (TEER) assays (Balharry et al. 2008; Burcham et al. 2014). The lung epithelium is a physical and immunological barrier to inhalable toxic agents, so barrier permeability is an important function of overall lung health (Brune et al. 2015). When lung epithelial cells are brought to ALI, TEER drops significantly, which normally indicates a more permeable barrier as discussed in one study (Kastner et al. 2013). However, when cells are cultured for about a week at ALI, depending on the cell type, ion channels can become active and cells become polarized (Harcourt and Haynes 2013; Ren et al. 2016). Both of these physiological changes can alter TEER values irrespective of membrane integrity (Harcourt and Haynes 2013; Kaufman et al. 2024). Immunofluorescence staining for junctional proteins, such as ZO-1 and E-cadherin and fluorescent dye permeability assays like FITC-dextran do not appear to fluctuate to the same degree as TEER when cells are cultured at ALI (Lv et al. 2024). Despite the physiological importance of barrier permeability in lung models, VOC-induced changes to barrier function are a less studied endpoint.

### Investigations into the mechanisms of VOC-induced respiratory toxicity

Of the 144 papers evaluated in this scoping review, approximately 20% employed intervention methods to validate potential VOC exposure mechanisms in the pulmonary epithelium. Although mechanisms were investigated across cell types, the majority of studies investigated acrolein-specific mechanisms of action. The role of ROS, induction of apoptosis, and involvement of the MAPK signaling pathways in mediating VOC exposure effects were the most investigated. All studies investigating mechanisms utilized the submerged cell culture method and a liquid VOC exposure medium.

An initial oxidative stress-related response was frequently observed following VOC exposure. This response encompassed either individual observations or combined observations of increased detection of ROS, depletion of GSH, and increased expression of antioxidant gene and protein expression. Notably, increased markers of oxidative stress-related endpoints is a common observation in submerged in vitro models investigating other forms of air pollution such as particulate matter, ozone, and wood smoke, as well as in in vivo studies of VOCs (Lino-dos-Santos-Franco et al. 2011; Cho et al. 2018; Tian et al. 2022; Abzhanova et al. 2024; Vitucci et al. 2024). To investigate the mechanistic role of this initial oxidative stress-related exposure response in mediating VOC-induced toxicity, studies utilized broad antioxidants, aldose reductase, and inhibition of specific redox-related signaling pathways, such as NRF2 and TRXR1. Pre- and/or concurrent treatment with these agents frequently decreased the observed VOC-induced toxicity, suggesting VOC-induced toxicity may be mediated through the induction of oxidative-stress-related alterations. A decrease in VOC-induced apoptosis, particularly when investigating chlorinated VOCs, was also observed following the inhibition of the specific apoptotic signaling pathways PPARy and p53. Antioxidant pretreatment decreased this VOC-induced apoptosis, further supporting the observation that initial oxidative stress-related endpoints are upstream of VOC-induced toxicity.

VOC exposure also perturbed the expression and secretion of various proinflammatory regulators, namely the cytokines IL-6, IL-8, MCP-1, and IL-11, and the prostaglandins PGE2 and PGF2a. Similar to above, the alteration of proinflammatory regulators is also observed following exposure to various other air pollutants (McCullough et al. 2014; Wang et al. 2017; Bowers et al. 2018; Janbazacyabar et al. 2022; Vitucci et al. 2024). MAPK signaling can mediate the transcription and secretion of pulmonary proinflammatory regulators (Wang et al. 2017; Saleem 2024). A small set of studies investigated the role of MAPK signaling in mediating the secretion of these proinflammatory regulators following VOC exposure. Through the utilization of chemical and small molecule inhibitors for specific MAPK signaling proteins, these studies observed that inhibition of MAPK signaling reversed the VOC-induced alterations to proinflammatory cytokine release, viability, and antioxidant expression. However, the role of MAPK signaling in mediating VOC-induced toxicity was variable between studies, suggesting it may be VOC or cell-type specific.

**Fig. 4.**
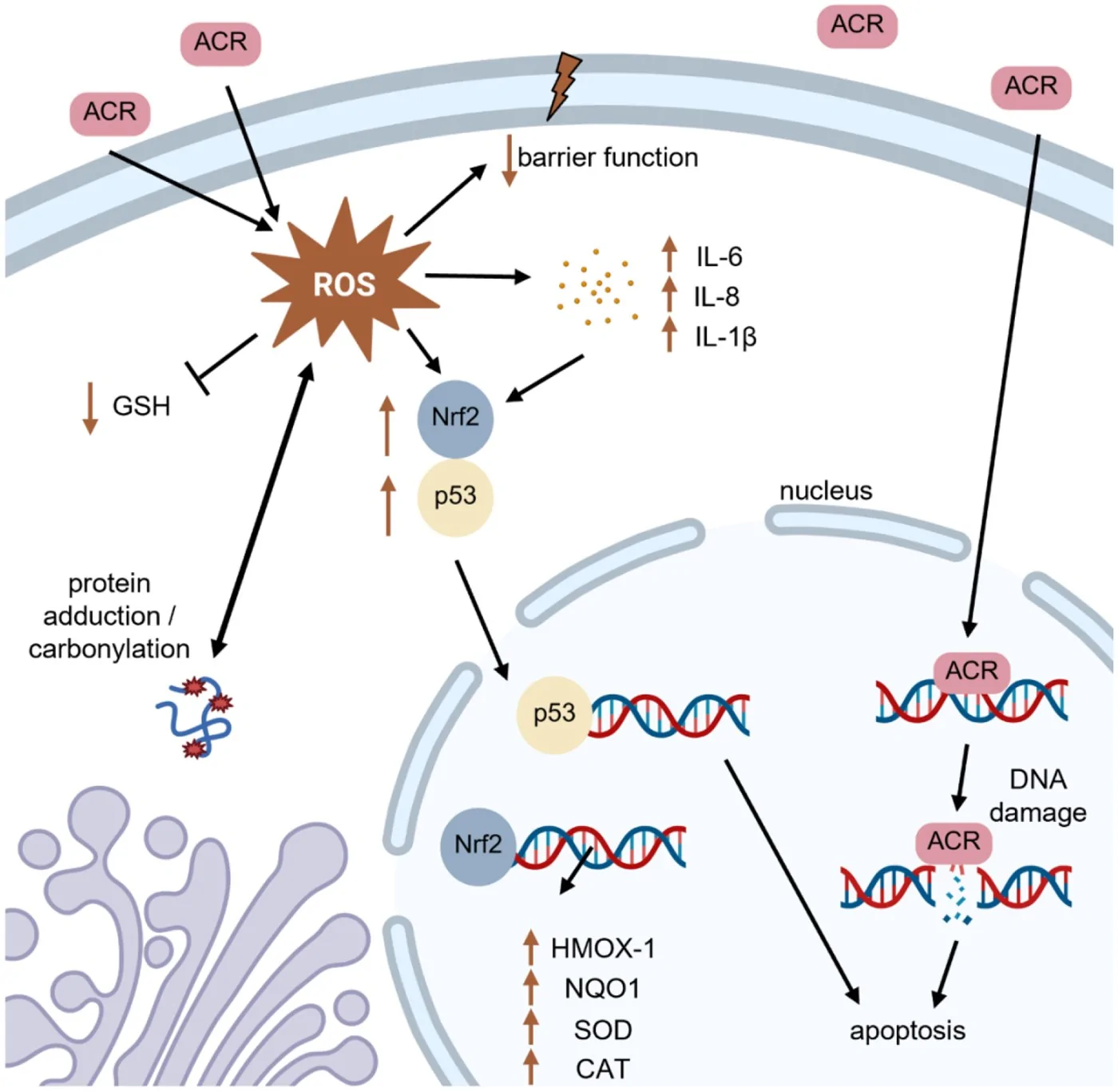
A summary of the toxicological effects and potential mechanisms of VOCs on human lung epithelial cells. Acrolein (ACR) is shown as a representative VOC. ROS generation is a frequently observed promoter of multiple downstream adverse effects including but not limited to changes in redox homeostasis, proinflammatory signaling, DNA adduct formation, and apoptosis.

Reactive aldehydes such as acrolein, can cause protein carbonylation, a protein adduct associated with oxidative stress and disruption of cellular processes (Wong et al. 2008; Suzuki et al. 2010; Ogbodo et al. 2022). Thus, the role by which VOCs, namely acrolein, can directly induce toxicity through protein adduct formation was also investigated. By utilizing small molecule inhibitors and carbonyl scavengers that minimized acrolein-induced protein carbonylation, these studies observed a decrease in acrolein-induced toxicity. Moreover, a decrease in ROS formation was also observed. These studies suggest that protein adduct formation may be an initiating step, upstream of the oxidative stress-related exposure responses, of VOC-induced toxicity, particularly of aldehyde VOCs as shown in Fig. 4.

Overall, oxidative stress-related alterations were a common observation following VOC exposures and consequentially, was the most common mechanism of VOC-induced toxicity investigated. Indeed, mechanistic studies suggest ROS formation and perturbation of redox homeostasis is a likely mediator of VOC-induced adverse exposure effects. However, more research is needed to investigate the universality of this observation across a larger distribution of VOCs and different lung epithelial cell types. A more robust investigation of this proposed mechanism in human, primary lung epithelial cells, and in ALI exposure models would also provide essential validation. Ultimately, additional investigations into the mechanisms of VOC-induced toxicity, including the induction of oxidative stress, proinflammatory signaling, and apoptosis are warranted to improve our understanding of the effects of VOC inhalation on the human lung.

## Conclusion and future directions

In contrast to the extensive literature on the pulmonary exposure effects of criteria air pollutants (carbon monoxide, lead, NOx, ozone, particulate matter, and sulfur dioxide), the literature on the pulmonary exposure effects of VOCs, a ubiquitous noncriteria air pollutant, is significantly lagging. This gap is further underscored by the paucity of studies investigating pulmonary VOC exposure effects and mechanisms in in vitro models. This review summarizes current literature utilizing in vitro models of the human lung epithelium to characterize the respiratory toxicity of VOCs. We found substantial heterogeneity in studied cell culture models, VOCs, and endpoints. Although there was a large range in VOC exposure methodologies, the majority of in vitro VOC exposure studies were conducted on pulmonary epithelial cells cultured in submerged models. The main biological endpoints assessed were related to cytotoxicity, oxidative stress, proinflammatory signaling, and apoptosis. Of the limited number of studies that investigated cellular VOC exposure mechanisms, epithelial oxidative stress was repeatedly noted to be upstream of the frequently observed altered proinflammatory signaling. Most of the research involved A549 cells and does not address the issue of population variability among individuals who differ in their susceptibility and sensitivity to VOC exposure. ALI improves the physiological characteristics of in vitro models and represents a more accurate inhalation exposure when cells are exposed to vapor rather than a liquid. However, ALI methodologies differ from lab to lab which creates a challenge when comparing studies of the same VOC, especially when the delivered dose is not validated. Ultimately, this review points out a general trend of creating increasingly complex in vitro systems to answer the same toxicological questions—at what dose does a significant effect occur? How long after exposure does this effect occur? What is the mechanism of action? Still, many of these systems have the same limitations: Nonexistent standardization, lack of an immune component, challenges with dose validation and uniformity, difficulty with chronic exposures, and biological endpoints that might or might not reflect physiological effects. As such, the authors pose the question of “How complex does an in vitro respiratory model have to be to answer the aforementioned toxicological queries?”.

The respiratory health effects of many VOCs have not been investigated and standardizing an ALI protocol, complete with quality controls, is profoundly needed within the field of inhalation toxicology. This synthesis of the current in vitro literature herein is a critical step in identifying and filling gaps in the assessment of VOC exposure effects and advancing our understanding of the mechanisms of VOC toxicity in the lung.

## Supplementary Material

kfag110_Supplementary_Data
