# Supplementary material for: In vitro approaches to assess respiratory toxicity from volatile organic compounds: an in-depth review
Source: Toxicol Sci. 2026 Aug 20;209(9):kfag110. doi: 10.1093/toxsci/kfag110 (PMC13557630; doi:10.1093/toxsci/kfag110)
Supplement: Supplementary file 1 [file kfag110_supplementary_data.docx]

**Supporting Information**

***In Vitro* Approaches to Assess Respiratory Toxicity from Volatile Organic Compounds:
An In-Depth Review**

Olivia C.G. Lampe^1^, Eva C.M. Vitucci^1^, Carolyn L. Cannon^2^, Kyle Holland ^3^, Margaret J. Foster^3^, Natalie M. Johnson^1^

*^1^ Department of Environmental and Occupational Health, Interdisciplinary Program in Toxicology, Texas A&M University, College Station, TX, 77843 USA*

*^2^ Department of Microbial Pathogenesis and Immunology, Texas A&M University, College Station, TX, 77843 USA*

*^3^ Medical Sciences Library, Texas A&M University, College Station, TX, 77843 USA*

July 2026

*Toxicological Sciences*

Corresponding author: Natalie Johnson

Email: nmjohnson@tamu.edu

No. of pages: 26 (without references)

No. of tables: 4

No. of figures: 1

**Supplemental Table 1**. VOC Search Terms for a representative database – MEDLINE

| **Medline Search Report and Search Terms** | | | |
| --- | --- | --- | --- |
| **Concept** | **Line** | **Query** | **Results** |
| lungs | 1 | exp Lung/ or exp Lung Diseases/ | 1502569 |
|  | 2 | (lung* or pulmo* or pneumo* or bronch* or alveol* or pleura*).ti,ab,kf. | 1830793 |
|  | 3 | or/1-2 | 2401502 |
| VOCs | 4 | exp Volatile Organic Compounds/ | 16262 |
|  | 5 | ("volatile organic compound*" or "semivolatile organic compound*" or "volatile organic chemical*" or "semivolatile organic chemical*" or VOC? or SVOC? or VVOC? or TVOC?).ti,ab,kf. | 29628 |
|  | 6 | exp Formaldehyde/ or Benzene/ or exp Toluene/ or Acrolein/ or exp Styrene/ or Xylenes/ or exp Glyoxal/ or Trichloroethylene/ or (formaldehyde or benzene or toluene or acrolein or chlorobenzene or styrene or xylene or methacrolein or propanal or butanal or pentanal or glyoxal or hexanal or trichloroethylene).ti,ab,kf,nm. | 177971 |
|  | 7 | or/4-6 | 208536 |
| "cell models" | 8 | exp Cells, Cultured/ | 1919126 |
|  | 9 | (((cell* or invitro or "in vitro" or tissue* or submerged) adj2 (model* or culture*)) or "culture model*" or "exposure system*" or ALI or "air liquid interface*").ti,ab,kf. | 514594 |
|  | 10 | or/8-9 | 2188620 |
|  | 11 | 3 and 7 and 10 | 1506 |

**Supplemental Table 2**. Literature search results summary. Acronyms for timepoints include (M = minutes, H = hours, D = days). *Studies (n=22) that utilized more than one cell line.

| **Study ID** | **Title** | **VOC(s)** | **Mixture?** | **Cell Type** | **Cell Culture Method** | **Exposure Medium** | **Dosage (ppm)** | **Time Points** | **Dose Validation Method if Applicable** | **Biological Endpoints** |
| --- | --- | --- | --- | --- | --- | --- | --- | --- | --- | --- |
| Agarwal 2013 | Metabolic shift in lung alveolar cell mitochondria following acrolein exposure. | acrolein | No | NCI-H441 | Submerged | Liquid | 0-6.72 | 4 H |  | Cytotoxicity; ROS generation; Metabolism; Other: Glycolysis |
| Al Zallouha 2017 | Usefulness of toxicological validation of VOCs catalytic degradation by air-liquid interface exposure system. | toluene | No | A549 | ALI | Vapor | 100; 1000 | 1 H |  | Cytotoxicity; Pro-Inflammatory Signaling; Metabolism |
| An 2019 | Low concentrations of FA exhibits the Hormesis effect by affecting cell division and the Warburg effect. | formaldehyde | No | 16HBE | Submerged | Liquid | 0.75-6 | 2 H |  | ROS generation; Cell proliferation; Other: Glycolysis |
| Anderson 2010 | Evaluation of dicarbonyls generated in a simulated indoor air environment using an in vitro exposure system. | methyl glyoxal; glyoxal; diacetyl; glutaraldehyde; 4-oxopentanal | No | A549 | ALI | Vapor | 15-65 | Exposure: 2 H; 4 H Collection: 8 H; 12 H; 24 H | GC-MS | Cytotoxicity; Pro-Inflammatory Signaling |
| Anderson 2013 | Toxicological analysis of limonene reaction products using an in vitro exposure system. | limonene;  ozone | Yes | A549 | ALI | Vapor | Limonene: 0.5; 20 Ozone: 0.1; 4 | Exposure: 1 H; 4 H Collection: 10 H; 12 H; 24 H | GC-MS | Pro-Inflammatory Signaling; Cell proliferation |
| Bakand 2005 | In vitro cytotoxicity testing of airborne formaldehyde collected in serum-free culture media. | formaldehyde | No | A549 | Submerged | Diffusion | 10 | 4 H | UV-VIS spectrophotometry | Cytotoxicity |
| Bakand 2006 | A novel in vitro exposure technique for toxicity testing of selected volatile organic compounds. | toluene;  xylene | No | A549 | ALI | Diffusion | 5350-16600 | 1 H |  | Cytotoxicity |
| Baldridge 2015 | Cellular RNA is chemically modified by exposure to air pollution mixtures. | ozone;  acrolein;  methacrolein | Yes | A549 | ALI | Vapor | acrolein (0.872); methacrolein (0.698) | 1 H |  | Cytotoxicity; Pro-Inflammatory Signaling; ROS generation |
| Balharry 2008 | An in vitro approach to assess the toxicity of inhaled tobacco smoke components: nicotine, cadmium, formaldehyde and urethane. | formaldehyde | No | EpiAirway-100 | ALI | Liquid | 30, 150, 225, 300, 451 | 24 H |  | Cytotoxicity; barrier function; Pro-Inflammatory Signaling; Other: H&E staining |
| Behar 2018 | Analytical and toxicological evaluation of flavor chemicals in electronic cigarette refill fluids. | cinnamaldehyde; menthol; benzyl alcohol; vanillin; eugenol;  p-anisaldehyde; ethyl cinnamate; maltol; ethyl maltol; triacetin; benzaldehyde; menthone | No | A549 | Submerged | Liquid | cinnamaldehyde (hPF: 0.2-200, A549: 20-10,000), menthol (10-6000), benzyl alcohol (3-2000), vanillin (3-3000), eugenol (1-600), p-anisaldehyde (0.7-500), ethyl cinnamate (0.6-600), maltol (0.7-250), ethyl maltol (0.3-300), triacetin (0.2-200), benzaldehyde (0.3-200), and menthone (0.2-100) | 48 H |  | Cytotoxicity |
| Bein 2020* | Albumin Protects Lung Cells against Acrolein Cytotoxicity and Acrolein-Adducted Albumin Increases Heme Oxygenase 1 Transcripts. | Acrolein | No | NCI-H441; primary human airway epithelial cells (HAEC) | Submerged | Liquid | 0.56; 1.12; 2.24; 3.36;4.49 | 72 H; |  | Cytotoxicity; Other: Antioxidant gene expression, albumin carbonylation |
| Billet 2010 | Benzene-induced mutational pattern in the tumour suppressor gene TP53 analysed by use of a functional assay, the functional analysis of separated alleles in yeast, in human lung cells. | benzene | No | A549 | Submerged | Liquid | 0.5 | 72 H |  | Cytotoxicity; Other: Mutagenicity |
| Binder 2022 | Construction of an In Vitro Air-Liquid Interface Exposure System to Assess the Toxicological Impact of Gas and Particle Phase of Semi-Volatile Organic Compounds. | dibutyl phthalate | No | A549 | ALI | Vapor | 0.15 | Exposure: 4 H Collection: 0 H; 24 H | Attempted LC-MS | Genotoxicity; Cytotoxicity |
| Biswal 2002 | Inhibition of cell proliferation and AP-1 activity by acrolein in human A549 lung adenocarcinoma cells due to thiol imbalance and covalent modifications. | acrolein | No | A549 | Submerged | Liquid | 2.8; 4.2 | 0.5 H |  | Cytotoxicity; Cell proliferation; Other: AP-1 and JNK expression and activity, total GSH concentration |
| Biswal 2003 | Modulation of benzo[a]pyrene-induced p53 DNA activity by acrolein. | acrolein; benzo[a]pyrene | Yes | A549 | Submerged | Liquid | BAP: 0.25; Acrolein: 2.8; 4.2 | BAP: 48 H; Acrolein: 0.5 H |  | ROS generation; Other: p53 Signaling |
| Borchers 1999 | Regulation of human airway mucins by acrolein and inflammatory mediators. | acrolein | No | NCI-H292 | Submerged | Liquid | 0.00024 - 2.442 | 4 H |  | Other: mucin transcription |
| Brostrom 2015* | Toluene diisocyanate: Induction of the autotaxin-lysophosphatidic acid axis and its association with airways symptoms. | toluene diisocyanate (TDI) | No | A549; 16HBE | Submerged | Liquid | 0.24-2445.0 | 0 - 24 H |  | Other: Lipid signaling |
| Brostrom 2018 | Toluene diisocyanate exposure and autotaxin-lysophosphatidic acid signalling. | toluene diisocyanate (TDI) | No | 16HBE | Submerged | Liquid | 0.000024 - 2.9 | 0.5 H |  | Pro-Inflammatory Signaling; Other: lipid secretion |
| Burcham 2007 | Intermolecular protein cross-linking during acrolein toxicity: efficacy of carbonyl scavengers as inhibitors of heat shock protein-90 cross-linking in A549 cells. | acrolein | No | A549 | Submerged | Liquid | 0.7 - 5.5 | 0.25 - 4 H |  | Cytotoxicity; Other: protein carbonylation, protein cross-linking |
| Burcham 2010b | Toxicity of smoke extracts towards A549 lung cells: role of acrolein and suppression by carbonyl scavengers. | acrolein | No | A549 | Submerged | Liquid | 0.7; 4.2 | 4 H |  | Cytotoxicity; Other: protein carbonylation |
| Burcham 2010a | Intermediate filament carbonylation during acute acrolein toxicity in A549 lung cells: functional consequences, chaperone redistribution, and protection by bisulfite. | acrolein | No | A549 | Submerged | Liquid | 0.7 - 5.5 | 0.5 H |  | Cytotoxicity; Other: protein carbonylation, protein phosphorylation, protein expression |
| Burcham 2012 | Chaperone heat shock protein 90 mobilization and hydralazine cytoprotection against acrolein-induced carbonyl stress. | Acrolein | No | A549 | Submerged | Liquid | 8.4; 4.2 | 3 H; 4 H |  | Cytotoxicity; Lipid Peroxidation; Other: protein adduct formation |
| Burcham 2014 | Airborne acrolein induces keratin-8 (Ser-73) hyperphosphorylation and intermediate filament ubiquitination in bronchiolar lung cell monolayers. | acrolein | No | Calu-3 | ALI | Vapor | ~3 - 29 | 0 - 24 H | derivatisation of 7-hydroxyquinoline from acrolein and fluorescence detection | Cytotoxicity; barrier function; Other: protein adduct formation, keratin signaling |
| Cheah 2013 | In vitro effects of aldehydes present in tobacco smoke on gene expression in human lung alveolar epithelial cells. | formaldehyde, acrolein; acetaldehyde | Yes | A549 | Submerged | Liquid | 0.008 - 63 | 1 H; 2 H; 4 H; 24 H |  | Cytotoxicity; Other: microarray |
| Chen 2013* | Cigarette smoke component acrolein modulates chromatin assembly by inhibiting histone acetylation. | acrolein | No | BEAS-2B, A549 | Submerged | Liquid | 0.28; 2.8; 5.6 | 0.5; 1; 2; 4 H |  | Cytotoxicity; Apoptosis; Other: histone modifications, chromatin accessibility |
| Chen 2017 | Regulation of Chromatin Assembly and Cell Transformation by Formaldehyde Exposure in Human Cells. | formaldehyde | No | BEAS-2B | Submerged | Liquid | 0; 3; 7.5; 15 | 6 H; 24 H; 48 H |  | Cytotoxicity; Metabolism; Cell proliferation; Other: Histone carbonylation chromatin accessibility |
| Chen 2002* | Possible involvement of glutathione and p53 in trichloroethylene- and perchloroethylene-induced lipid peroxidation and apoptosis in human lung cancer cells. | trichloroethylene (TCE); perchloroethylene (PERC) | No | NCI-H460; NCI-HI299 | Submerged | Diffusion | 232.5; 465.1; 930.2 PERC 930.2; 1860.5; 3720.9 TCE | 24 H | purge and trap GC-MS | Apoptosis; Lipid Peroxidation; ROS generation; Cell proliferation |
| Cheng 2023 | Oxidative Stress Effects of Multiple Pollutants in an Indoor Environment on Human Bronchial Epithelial Cells. | benzene, toluene, xylenes | No | BEAS-2B | Submerged | Liquid | 0.015- 0.300 x 10^-3^ | 1; 6; 12; 24; 48; 72 H | GC-MS | Cytotoxicity; Apoptosis; Pro-Inflammatory Signaling; ROS generation; Metabolism; Other: Mitochondrial membrane potential |
| Chiung 2009 | Toluene diisocyanate elevates cytosolic calcium concentration, mediator secretion and cytokine expression in airway cell lines and peripheral WBC | toluene diisocyanate | No | H1355 | Submerged | Liquid | 0.17 - 170 | 5 M |  |  |
| Cho 2015 | Hexanal-induced changes in miRNA-mRNA interactions in A549 human alveolar epithelial cells | hexanal | No | A549 | Submerged | Liquid | 80 | 48 H |  | Other: Calcium-signaling pathway, miRNA expression |
| Cho 2018 | DNA Methylome Analysis of Saturated Aliphatic Aldehydes in Pulmonary Toxicity. | propanal; butanal; pentanal; hexanal; heptanal; octanal; nonanal | No | A549 | Submerged | Liquid | 31.68-654.12 | 48 H |  | Genotoxicity; Cytotoxicity |
| Choi 2004 | Increased levels of IgG to cytokeratin 19 in sera of patients with toluene diisocyanate-induced asthma. | toluene diisocyanate | No | BEAS-2B | Submerged | Liquid | 50 | 24 H |  | Pro-Inflammatory Signaling; Other: expression of cytokeratin 19 (CK19) protein |
| Choi 2011 | Inhibition of acrolein-stimulated MUC5AC expression by Platycodon grandiflorum root-derived saponin in A549 cells. | Acrolein | No | A549 | Submerged | Liquid | 0-0.00168 | 0; 2; 4; 8; 12; 24 H |  | Cytotoxicity; Pro-Inflammatory Signaling; ROS generation; Other: mucin production |
| Cui 2016 | Formaldehyde impairs transepithelial sodium transport. | formaldehyde | No | NCI-H441 | Submerged | Liquid | 6000 | 0; 0.5; 1.5; 2; 6; 24; 48 H |  | barrier function; ROS generation; Other: short circuit current |
| Deshmukh 2005* | Metalloproteinases mediate mucin 5AC expression by epidermal growth factor receptor activation. | Acrolein | No | NCI-H292; NHBE | Submerged | Liquid | 0.0006-5.6 | 4 H |  | Pro-Inflammatory Signaling; ROS generation; Other: metalloproteinase expression |
| Doyle 2007 | Atmospheric photochemical transformations enhance 1,3-butadiene-induced inflammatory responses in human epithelial cells: The role of ozone and other photochemical degradation products | 1,3-butadiene; acrolein; acetaldehyde; formaldehyde; furan; ozone | Yes | A549 | ALI | Vapor | 0.087-0.199 | 5 H | GC-FID; GC-ECD; GC-MS | Cytotoxicity; Pro-Inflammatory Signaling |
| Du 2004 | Safrole oxide induces apoptosis in A549 human lung cancer cells. | safrole oxide | No | A549 | Submerged | Liquid | 0; 20; 40; 80 | 24 H; 48 H |  | Cytotoxicity; Apoptosis; Cell proliferation |
| Du 2006 | Safrole oxide induces apoptosis by up-regulating Fas and FasL instead of integrin beta4 in A549 human lung cancer cells. | safrole oxide | No | A549 | Submerged | Liquid | 40 | 24 H |  | Apoptosis |
| Duistermaat 2025 | Towards prospective identification of respiratory sensitizers: effects of piperazine, chloramine-T, and toluene-diisocyanate in an air-liquid interface model comprising human bronchial epithelial cells. | toluene diisocyanate; piperazine;  chloramine-T | No | Calu-3 | ALI | Vapor | 0.23-25.6 | 2 H/D for 3 D | weight (i.e the VOC bottle was weighed before and after exposure) | Cytotoxicity; barrier function; Pro-Inflammatory Signaling; Metabolism |
| Dwivedi 2018* | Inflammatory effects of acrolein, crotonaldehyde and hexanal vapors on human primary bronchial epithelial cells cultured at air-liquid interface. | acrolein; crotonaldehyde; hexanal | No | co-culture pHBEs and MRC-5 (ALI); pHBEs (submerged) | ALI | Liquid; Vapor | 0.043-54 | Exposure: 30 M Collection: 6 H; 8 H; 24 H | GC-FID | Cytotoxicity; Pro-Inflammatory Signaling; Other: metalloproteinase expression |
| Ebersviller 2012 | Gaseous VOCs rapidly modify particulate matter and its biological effects - Part 1: Simple VOCs and model PM. | acrolein;  p-tolualdehyde | Yes | A549 | ALI | Vapor | 2.0; 1.0 | Exposure: 4 H Collection: 9 H | GC-FID; GC-ECD | Cytotoxicity; Pro-Inflammatory Signaling |
| Feltens 2010 | Chlorobenzene induces oxidative stress in human lung epithelial cells in vitro. | chlorobenzene | No | A549 | ALI | Diffusion | 0.22; 0.022; 2.2 | 2 H; 4 H; 9 H; 24 H |  | Pro-Inflammatory Signaling; ROS generation |
| Feng 2006 | Acrolein is a major cigarette-related lung cancer agent: Preferential binding at p53 mutational hotspots and inhibition of DNA repair. | acrolein | No | NHBE | Submerged | Liquid | 0-5.6 | 6 H |  | Genotoxicity |
| Fischader 2008 | Release of MCP-1 and IL-8 from lung epithelial cells exposed to volatile organic compounds. | n-nonane; n-decane; n-undecane; n-dodecane, n-tridecane; methylcyclopentane; styrene; m-xylene; chlorobenzene | Yes | A549 | Submerged | Diffusion | 0.00002-23,500 | 20 H |  | Cytotoxicity; Pro-Inflammatory Signaling |
| Gonzalez-Rivera 2020 | RNA oxidation in chromatin modification and DNA-damage response following exposure to formaldehyde. | formaldehyde | No | BEAS-2B | ALI | Vapor | 1 | Exposure: 2 H Collection: 6 H | chemical ionization mass spectrometry (CIMS) | Cytotoxicity; Other: 8-oxoG enriched transcriptomics |
| Gostner 2016 | Cellular reactions to long-term volatile organic compound (VOC) exposures. | formaldehyde | No | A549 | ALI | Vapor | 0.1; 0,5 | 3 D | Aerolaser 4021 | Cytotoxicity; Metabolism |
| Giuliano 2009 | Effects of low concentrations of benzene on human lung cells in vitro. | benzene | No | A549 | Submerged | Liquid | 0.0008-0.08 | 0; 6, 24, 48, 54; 72; 96 H |  | Genotoxicity; Cytotoxicity; Other: expression of metalloproteinases |
| Gminski 2010 | Cytotoxicity and genotoxicity in human lung epithelial A549 cells caused by airborne volatile organic compounds emitted from pine wood and oriented strand boards. | delta-3-carene;  alpha-pinene; beta-pinene; camphene: limonene; hexanal;  2-heptenal; 2-octenal; pentanal | Yes | A549 | ALI | Vapor | 0.16-448 | 1 H | GC-MS | Genotoxicity; Cytotoxicity |
| Grafstrom 1988 | Pathobiological effects of acrolein in cultured human bronchial epithelial cells. | Acrolein | No | pHBEs | Submerged | Liquid | 0-16.8 | 1 H; 3 H |  | Cytotoxicity; ROS generation; Cell proliferation; Other: plasminogen activator activity |
| Han 2023 | Chronic Exposure to TDI Induces Cell Migration and Invasion via TGF-beta1 Signal Transduction. | toluene diisocyanate (TDI) | No | BEAS-2B | Submerged | Liquid | 0.17-87 | 1 D; 3 D; 7 D; 2 months |  | Cytotoxicity; Apoptosis; Pro-Inflammatory Signaling; Metabolism; Other: cell migration, expression of epithelial-mesenchymal transition proteins |
| Horton 1997 | Relationships between cell density, glutathione and proliferation of A549 human lung adenocarcinoma cells treated with acrolein. | Acrolein | No | A549 | Submerged | Liquid | 0.01 - 1 | 24 - 96 H |  | Cytotoxicity; Cell proliferation; Other: total cellular glutathione levels, cellular stress response |
| Horton 1999 | Acrolein causes inhibitor kappaB-independent decreases in nuclear factor kappaB activation in human lung adenocarcinoma (A549) cells. | Acrolein | No | A549 | Submerged | Liquid | 0.38 | 30 M; 2 H; 4 H; 8 H; 12 H; 24 H |  | Pro-Inflammatory Signaling; ROS generation; Cell proliferation |
| Jiang 2020 | The scavenging capacity of gamma-aminobutyric acid for acrolein and the cytotoxicity of the formed adduct. | Acrolein | No | human bronchial epithelial cell line | Submerged | Liquid | 0.1 - 10 | 24 H; 48 H |  | Cytotoxicity; Apoptosis; ROS generation |
| Johanson 2020 | Analysis of Acrolein Exposure Induced Pulmonary Response in Seven Inbred Mouse Strains and Human Primary Bronchial Epithelial Cells Cultured at Air-Liquid Interface. | Acrolein | No | pHBEs | ALI | Vapor | 0.05; 0.1; 0.2; 0.5 | 24 H | GC-MS | Pro-Inflammatory Signaling |
| Jude 2016* | Formaldehyde Induces Rho-Associated Kinase Activity to Evoke Airway Hyperresponsiveness. | Formaldehyde | No | pHBEs; SAECs; human precision cut lung slices | ALI | Vapor | 0.2; 0.8; 2 | 24 H | passive formaldehyde monitors | Cytotoxicity; Pro-Inflammatory Signaling; Other: Oxidative stress signaling, airway responsiveness |
| Kastner 2011* | Formaldehyde interferes with airway epithelium integrity and functions in a dose- and time-dependent manner. | formaldehyde | No | Calu-3, 16HBE | Submerged | Liquid | 2.1 - 210 | 24 H |  | Cytotoxicity; Apoptosis; barrier function; Pro-Inflammatory Signaling; ROS generation |
| Kastner 2013 | A dynamic system for single and repeated exposure of airway epithelial cells to gaseous pollutants | formaldehyde;  nitrogen dioxide | Yes | Calu-3 | ALI | Vapor | 0.16 - formaldehyde 0.425 - nitrogen dioxide | Exposure: 30 M Collection: 24 H | 2,4-DNPH derivative technique | Cytotoxicity; barrier function; Pro-Inflammatory Signaling |
| Kim 2010* | Toluene diisocyanate (TDI) regulates haem oxygenase-1/ferritin expression: implications for toluene diisocyanate-induced asthma. | toluene diisocyanate | No | A549; BEAS-2B | Submerged | Liquid | 44; 88; 175 | 12; 24; 48 H |  | Other: oxidative stress signaling, ferritin signaling, MAPK signaling |
| Kim 2012 | Gene expression profiles of human lung epithelial cells exposed to toluene | Toluene | No | BEAS-2B | Submerged | Liquid; Diffusion | 27.6-441.6 | 6 H; 24 H |  | Pro-Inflammatory Signaling |
| Kim 2017 | Toluene diisocyanate exposure induces airway inflammation of bronchial epithelial cells via the activation of transient receptor potential melastatin 8. | toluene diisocyanate | No | BEAS-2B | Submerged | Liquid | 43.5-174 | 10 M; 1 H; 3 H |  | Pro-Inflammatory Signaling; Other: expression of transient receptor potential melastatin 8 (TRPM8) |
| Kim 2022 | Exposure of Toluene Diisocyanate Induces DUSP6 and p53 through Activation of TRPA1 Receptor. | toluene diisocyanate (TDI) | No | BEAS-2B | Submerged | Liquid | 0.017 - 850 | 4, 24, 72, 168 H |  | Cytotoxicity; Apoptosis; Other: MAPK signaling, p53 signaling |
| Komori 2008 | Development of an in vitro batch-type closed gas exposure device with an alveolar epithelial cell line, A549, for toxicity evaluations of gaseous compounds. | benzene; trichloroethylene; acetone | No | A549 | Submerged | Liquid | 30,000; 100,000 | 48 H |  | Cytotoxicity |
| Lange 1999 | Toluene diisocyanate colocalizes with tubulin on cilia of differentiated human airway epithelial cells. | toluene diisocyanate | No | pHBEs | ALI | Diffusion | 0.01-1 | 30 M | HPLC; Autostep gas analyzer | Genotoxicity; Cytotoxicity; barrier function; Other: TEM for structural changes |
| Lantz 2001 | Rapid reduction of intracellular glutathione in human bronchial epithelial cells exposed to occupational levels of toluene diisocyanate. | toluene diisocyanate | No | pHBEs | ALI | Diffusion | 0.02; 0.05; 0.1 | 5; 15; 30 M | Autostep continuous toxic gas analyzer | Cytotoxicity; ROS generation; Other: cellular esterase activity |
| Le 2025 | Characterizing the Pulmonary Toxicity and Potential Mutagenicity of Formaldehyde Fumes in a Human Bronchial Epithelial Tissue Model. | Formaldehyde | No | pHBEs | ALI | Vapor | 7.5; 15; 30 | Exposure: 4 H/D Collection: 5 D; 10 D; 15 D; 20 D; 48 D | HPLC-PDA | Genotoxicity; Cytotoxicity; Apoptosis; barrier function; Pro-Inflammatory Signaling; Cell proliferation; Other: ciliary function, cyst formation |
| Lecureur 2012* | MAPK- and PKC/CREB-dependent induction of interleukin-11 by the environmental contaminant formaldehyde in human bronchial epithelial cells. | formaldehyde | No | A549; BEAS-2B | Submerged | Liquid | 1.5; 3; 4.5; 6 | 6 H; 16 H; 24 H |  | Pro-Inflammatory Signaling; Metabolism |
| Lee 2012 | Gene expression profiling of low dose exposure of saturated aliphatic aldehydes in A549 human alveolar epithelial cells | propanal; butanal; pentanal; hexanal; octanal; nonanal; heptanal | No | A549 | Submerged | Liquid | propanal - 34.85; pentanal - 64.60; hexanal - 10.02; heptanal - 22.84; octanal - 19.23; butanal - 295.65; nonanal - 29.87 | 48 H |  | Cytotoxicity; Metabolism; Cell proliferation |
| Lehmann 2008 | In vitro models for the assessment of inflammatory and immuno-modulatory effects of the volatile organic compound chlorobenzene. | chlorobenzene | No | A549 | ALI | Vapor | 0-19945 | 20 H |  | Cytotoxicity; Pro-Inflammatory Signaling |
| Lim 2010 | Formaldehyde induces apoptosis through decreased Prx 2 via p38 MAPK in lung epithelial cells. | formaldehyde | No | A549 | Submerged | Liquid | 3, 30, 300, 3000 | 0.5 - 48 H |  | Cytotoxicity; Apoptosis; Lipid Peroxidation; Other: Oxidative stress signaling, MAPK signaling |
| Lim 2013 | The ER stress-mediated decrease in DDAH1 expression is involved in formaldehyde-induced apoptosis in lung epithelial cells. | formaldehyde | No | A549 | Submerged | Liquid | 0; 3; 30; 300; 3000 | 0.5; 1; 4; 24; 48 H |  | Cytotoxicity; Apoptosis; Other: dimethylarginine dimethylaminohydrolase expression, ER stress marker protein expression |
| Lim 2021 | Prediction of acute inhalation toxicity using cytotoxicity data from human lung epithelial cell lines. | acrolein; chloropicrin; glutaral; ammonia; formaldehyde; glyoxal; hydrogen peroxide; dimethyl sulfate; dimethyltin dichloride; 1,1,2,2-tetrabromoethane; 1,1,2,2- tetrachloroethane;  2,2-bioxirane; epichlorohydrin; furfuryl alcohol; glycidol; octhilinone;  2-furaldehyde;  4-chloro-ocresol; dipropylamine; styrene; 1,1,2-trichloroethane; 1,2,3-trichloropropane; 1,3-dichloropropene; 2-dimethylaminoethanol; methacrylic acid | No | A549 | Submerged | Liquid | 0-5000 ug/mL* | 24 H |  | Cytotoxicity |
| Lin 2014 | Application of chemical vapor generation systems to deliver constant gas concentrations for in vitro exposure to volatile organic compounds | acrolein;  methacrolein | No | A549 | ALI | Vapor | acrolein – 0.23-2.37 methacrolein – 0.68-10.7 | Exposure: 4 H Collection: 9 H | GC-FID | Cytotoxicity; Pro-Inflammatory Signaling |
| Liu 2010 | Gene expression profile and cytotoxicity of human bronchial epithelial cells exposed to crotonaldehyde. | crotonaldehyde | No | BEAS-2B | Submerged | Liquid | 0-11.2 | 3 H; 6 H |  | Cytotoxicity; Pro-Inflammatory Signaling; Metabolism; Other: transcriptomics, cell cycle arrest |
| Liu 2011 | Effects of long-term low-dose formaldehyde exposure on global genomic hypomethylation in 16HBE cells. | formaldehyde | No | 16HBE | Submerged | Liquid | 0.3 | Exposure: 24 H Collection: 6 W; 12 W; 24 W |  | Genotoxicity |
| Liu 2013 | Hanging drop: an in vitro air toxic exposure model using human lung cells in 2D and 3D structures. | benzene | No | A549 | Submerged | Diffusion | 25-500 | 1 H; 24 H; 20 D |  | Genotoxicity; Cytotoxicity; Pro-Inflammatory Signaling |
| Liu 2014 | Mixture effects of benzene, toluene, ethylbenzene, and xylenes (BTEX) on lung carcinoma cells via a hanging drop air exposure system. | benzene; toluene; ethylbenzene; xylenes | Yes | A549 | Submerged | Hanging Drop | 9957-27518 | 1 H; 24 H | GC-MS | Cytotoxicity |
| Liu 2015 | BTEX in vitro exposure tool using human lung cells: Trips and gains | benzene; toluene; ethylbenzene; xylenes | No | A549 | Submerged | Liquid; Diffusion; Hanging Drop | approx 390-8493 | 1 H, 6 H, 12 H, 24 H, 48 H, 72 H |  | Cytotoxicity |
| Liu 2022 | Toxicity mechanism of acrolein on DNA damage and apoptosis in BEAS-2B cells: Insights from cell biology and molecular docking analyses. | acrolein | No | BEAS-2B | Submerged | Liquid | 1.12; 2.24; 4.48; 8.96 | 3; 6; 12; 24 H |  | Genotoxicity; Cytotoxicity; Apoptosis; ROS generation |
| Lv 2024 | GLUT1 mediates bronchial epithelial E-cadherin disruption in TDI-induced steroid-insensitive asthma. | toluene diisocyanate (TDI) | No | pHBEs | ALI | Liquid | 17.4 | 30 M |  | Cytotoxicity; barrier function |
| Mascelloni 2015 | Study of gaseous benzene effects upon A549 lung epithelial cells using a novel exposure system. | benzene | No | A549 | ALI | Vapor | 0.03; 0.1; 0.3 | 2 H |  | Genotoxicity; ROS generation |
| Mattoli 1991 | Time course of IL1 and IL6 synthesis and release in human bronchial epithelial cell cultures exposed to toluene diisocyanate. | toluene diisocyanate | No | pHBEs | ALI | Vapor | 0.008; 0.018 | Exposure: 30 M Collection: 6 H; 24 H; 3 D; 6 D | Toxic gas monitor (MDA model 7005) | Cytotoxicity; Pro-Inflammatory Signaling; Cell proliferation |
| Meausoone 2019 | In vitro toxicological evaluation of emissions from catalytic oxidation removal of industrial VOCs by air/liquid interface (ALI) exposure system in repeated mode. | toluene | No | BEAS-2B | ALI | Vapor | 100; 1000 | Exposure: 1 H Collection: 1 D; 3 D; 5 D | GC-MS | Cytotoxicity; Pro-Inflammatory Signaling; Metabolism; Cell proliferation |
| Meausoone 2021 | Toxicological responses of BEAS-2B cells to repeated exposures to benzene, toluene, m-xylene, and mesitylene using air-liquid interface method. | benzene;  m-xylene;  toluene;  mesitylene | No | BEAS-2B | ALI | Vapor | 20; 100 | Exposure: 1 H Collection: 0 H; 3 D; 5 D |  | Pro-Inflammatory Signaling; Metabolism |
| Moretto 2012* | Cigarette smoke and its component acrolein augment IL-8/CXCL8 mRNA stability via p38 MAPK/MK2 signaling in human pulmonary cells. | Acrolein | No | SAECs; NHBEs | Submerged | Liquid | 0.56-3.36 | 0; 2; 4; 8; 18; 24 H |  | Cytotoxicity; Pro-Inflammatory Signaling |
| Mogel 2011 | The aromatic volatile organic compounds toluene, benzene and styrene induce COX-2 and prostaglandins in human lung epithelial cells via oxidative stress and p38 MAPK activation. | toluene;  benzene;  styrene | No | A549 | ALI | Diffusion | 0.02-2874152 | 1 H; 5 H; 8 H; 24 H | SPME and GC-MS | Cytotoxicity; ROS generation; Metabolism |
| Morbt 2009 | Proteome changes in human bronchoalveolar cells following styrene exposure indicate involvement of oxidative stress in the molecular-response mechanism. | Styrene | No | A549 | ALI | Diffusion | 0.22-2155 | 2 H; 24 H | GC-FID | Cytotoxicity; Pro-Inflammatory Signaling; ROS generation; Metabolism |
| Morbt 2011 | Chlorinated benzenes cause concomitantly oxidative stress and induction of apoptotic markers in lung epithelial cells (A549) at nonacute toxic concentrations. | chlorobenzene;  1,2-dichlorobenzene | No | A549 | ALI | Vapor | 1.53; 19.9 | 24 H | GC-FID | Cytotoxicity; Apoptosis; ROS generation; Metabolism |
| Murugesan 2013 | Subtoxic and toxic concentrations of benzene and toluene induce Nrf2-mediated antioxidative stress response and affect the central carbon metabolism in lung epithelial cells A549. | benzene;  toluene | No | A549 | Submerged | Diffusion | 28.7; 287 | 24 H |  | ROS generation; Metabolism |
| Myers 2009 | The effects of acrolein on peroxiredoxins, thioredoxins, and thioredoxin reductase in human bronchial epithelial cells. | acrolein | No | BEAS-2B | Submerged | Liquid | 0-1.4 | 30 M |  | Other: redox state of proteins |
| Najera-Martinez 2012 | Halomethane-induced cytotoxicity and cell proliferation in human lung MRC-5 fibroblasts and NL20-TA epithelial cells. | bromodichloromethane; trichloromethane; dichloromethane | No | NL20-TA | Submerged | Liquid | (8.4x10^-14^) - 16.38 | Exposure: 24 H Collection: 48 H |  | Cytotoxicity; ROS generation; Metabolism; Cell proliferation |
| Nardini 2002 | Acrolein-induced cytotoxicity in cultured human bronchial epithelial cells. Modulation by alpha-tocopherol and ascorbic acid. | acrolein | No | HBE1 | Submerged | Liquid | 0; 0.28; 0.56; 1.12; 1.4 | Exposure: 30 M Collection: 0; 12; 18; 24 H |  | Cytotoxicity; Apoptosis; ROS generation |
| Neuss 2008 | Further characterization of the genotoxicity of formaldehyde in vitro by the sister chromatid exchange test and co-cultivation experiments. | formaldehyde | No | A549 | Submerged | Liquid | 1.5; 3; 6; 9 | Exposure: 1 H Collection: 48 H |  | Genotoxicity |
| Ortega-Atienza 2015* | Proteasome activity is important for replication recovery, CHK1 phosphorylation and prevention of G2 arrest after low-dose formaldehyde. | formaldehyde | No | A549; NCI-H460 | Submerged | Liquid | 0; 1.5; 3 | Exposure: 3 H Collection: 0 H; 72 H |  | Genotoxicity; Cytotoxicity |
| Pariselli 2009a | Effects of toluene and benzene air mixtures on human lung cells (A549). | toluene; benzene | Yes | A549 | ALI | Vapor | 0.25; 0.28 | Exposure: 1 H Collection: 30 M; 3 H; 24 H | GC-MS | Genotoxicity; Cytotoxicity; ROS generation |
| Pariselli 2009b | An optimized method for in vitro exposure of human derived lung cells to volatile chemicals. | benzene;  toluene;  formaldehyde | No | A549 | ALI | Vapor | 0.1; 0.2; 0.3; 0.6 | Exposure: 2 H; Collection: 24 H | GC-MS; HPLC | Cytotoxicity; Pro-Inflammatory Signaling; ROS generation |
| Park 2020 | Combination Therapy with Cinnamaldehyde and Hyperthermia Induces Apoptosis of A549 Non-Small Cell Lung Carcinoma Cells via Regulation of Reactive Oxygen Species and Mitogen-Activated Protein Kinase Family. | cinnamaldehyde | No | A549 | Submerged | Liquid | 20; 27 | 4 H; 24 H; 48 H |  | Cytotoxicity; Apoptosis; ROS generation; Cell proliferation |
| Persoz 2010 | An in vitro model to evaluate the inflammatory response after gaseous formaldehyde exposure of lung epithelial cells. | formaldehyde | No | A549 | ALI | Vapor | 0.02; 0.04; 0.06 | Exposure: 30 M; 60 M Collection: 24 H | HPLC | Cytotoxicity; Pro-Inflammatory Signaling |
| Persoz 2011 | Sequential air-liquid exposure of human respiratory cells to chemical and biological pollutants. | formaldehyde | No | A549 | ALI | Vapor | 0.04 | 14 H |  | Cytotoxicity; Pro-Inflammatory Signaling |
| Persoz 2012* | Inflammatory response modulation of airway epithelial cells exposed to formaldehyde | formaldehyde | No | A549; BEAS-2B | ALI | Vapor | 0.037 | Exposure: 30 M Collection: 24 H |  | Pro-Inflammatory Signaling |
| Pokharel 2008 | Inhibition of acrolein-stimulated MUC5AC production by fucoidan in human bronchial epithelial cells. | acrolein | No | NCI-H292 | Submerged | Liquid | 0-0.001 | 4 H |  | Other: mucin gene expression |
| Rager 2010 | Formaldehyde exposure alters microRNA expression profiles in human lung cells | formaldehyde | No | A549 | ALI | Vapor | 1 | 9 H |  | Cytotoxicity; Pro-Inflammatory Signaling; Other: miRNA expression |
| Rager 2011 | Epigenetic changes induced by air toxics: formaldehyde exposure alters miRNA expression profiles in human lung cells. | Formaldehyde | No | A549 | ALI | Vapor | 1 | Exposure: 4 H Collection: 9 H |  | Cytotoxicity; Pro-Inflammatory Signaling; Other: miRNA expression |
| Randall 2013 | Acrolein-induced activation of mitogen-activated protein kinase signaling is mediated by alkylation of thioredoxin reductase and thioredoxin 1. | Acrolein | No | HBE1 | Submerged | Liquid | 0.17-1.68 | Exposure: 30 M Collection: 24 H |  | Cytotoxicity; Pro-Inflammatory Signaling; ROS generation; Other: redox state of proteins |
| Randall 2013 | Protein alkylation by the alpha,beta-unsaturated aldehyde acrolein. A reversible mechanism of electrophile signaling?. | Acrolein | No | HBE1 | Submerged | Liquid | 1.7 | 0.5 - 24 H |  | Other: oxidative stress signaling |
| Roder-Stolinski 2008 | Styrene induces an inflammatory response in human lung epithelial cells via oxidative stress and NF-kappaB activation. | Styrene | No | A549 | ALI | Diffusion | 0.00002-215554489 | 1 H; 20 H |  | Cytotoxicity; Pro-Inflammatory Signaling; ROS generation |
| Roder-Stolinski 2008 | Chlorobenzene induces the NF-kappa B and p38 MAP kinase pathways in lung epithelial cells. | chlorobenzene | No | A549 | ALI | Diffusion | 0.00002-19950 | 1 H; 20 H |  | Cytotoxicity; Apoptosis; Pro-Inflammatory Signaling |
| Roy 2009 | Acrolein induces a cellular stress response and triggers mitochondrial apoptosis in A549 cells. | Acrolein | No | A549 | Submerged | Liquid | 0.056-1.51 | 30 M; 1 H; 2 H |  | Cytotoxicity; Apoptosis; ROS generation; Other: mitochondrial membrane potential, catalase activity, cell cycle analysis |
| Roy 2010 | Acrolein induces apoptosis through the death receptor pathway in A549 lung cells: role of p53. | Acrolein | No | A549 | Submerged | Liquid | 0.056-1.51 | 30 M; 1 H; 2 H |  | Apoptosis; ROS generation; Other: Mitochondrial membrane potential |
| Sakolish 2022 | A Model of Human Small Airway on a Chip for Studies of Subacute Effects of Inhalation Toxicants | Iodomethane | No | SAECs | ALI | Vapor | 0; 500; 1000 | Exposure: 5 H Collection: 24 H |  | Cytotoxicity; barrier function |
| Saladino 1985 | Effects of formaldehyde, acetaldehyde, benzoyl peroxide, and hydrogen peroxide on cultured normal human bronchial epithelial cells. | formaldehyde; acetaldehyde | No | pHBEs | Submerged | Liquid | formaldehyde: 6.3 acetaldehyde: 1320 | 6 H |  | Genotoxicity; Cell proliferation; Other: aryl hydrocarbon hydroxylase activity, arachidonic and choline release |
| Sarkar 2019 | Response of DNA damage genes in acrolein-treated lung adenocarcinoma cells. | acrolein | No | A549 | Submerged | Liquid | 2.8 | 2; 4; 6; 12; 24 H |  | Cytotoxicity; Apoptosis; Cell proliferation; Other: DNA damage signaling |
| Sauer 2013* | In vivo-in vitro comparison of acute respiratory tract toxicity using human 3D airway epithelial models and human A549 and murine 3T3 monolayer cell systems | methyl methacrylate; glutaraldehyde; aniline; acetic anhydride; isophorone diisocyanate; toluene diisocyanate; formaldehyde; acetone; ethanol; N,N-dimethyl formamide; trimellitic anhydride | No | EpiAirway; MucilAir; A549 | Submerged | Liquid | 18 - 722,007 | 24 H |  | Cytotoxicity; barrier function |
| Sayyed 2022 | Acute cytotoxicity, genotoxicity, and apoptosis induced by petroleum VOC emissions in A549 cell line. | gasoline | Yes | A549 | ALI | Diffusion | 0-735 | Exposure: 1 H Collection: 0 H; 24 H |  | Genotoxicity; Cytotoxicity; Apoptosis; Pro-Inflammatory Signaling; Cell proliferation |
| Sheets 2004* | Benzene metabolism in human lung cell lines BEAS-2B and A549 and cells overexpressing CYP2F1. | benzene | No | BEAS-2b; A549 | Submerged | Liquid | 15.6 | 45 M |  | Metabolism |
| Song 2014 | Analysis of microRNA and mRNA expression profiles highlights alterations in modulation of the MAPK pathway under octanal exposure | octanal | No | A549 | Submerged | Liquid | 19.5 - 260 | 48 H |  | Cytotoxicity; Other: miRNA expression, mRNA microarray |
| Song 2014 | Transcriptome profile analysis of saturated aliphatic aldehydes reveals carbon number-specific molecules involved in pulmonary toxicity. | propanal; butanal; pentanal; hexanal; heptanal; octanal | No | A549 | Submerged | Liquid | 68.4-331.2 | 48 H |  | Cytotoxicity; Other: transcriptomics |
| Song 2015 | Integrated analysis of microRNA and mRNA expression profiles highlights aldehyde-induced inflammatory responses in cells relevant for lung toxicity | propanal;  butanal;  pentanal | No | A549 | Submerged | Liquid | propanal - 35; 70; 145 butanal - 295; 310; 332 pentanal - 75; 100; 170 | 48 H |  | Cytotoxicity; Pro-Inflammatory Signaling; Other: miRNA expression |
| Speen 2022* | Benchmark Dose Modeling Approaches for Volatile Organic Chemicals Using a Novel Air-Liquid Interface In Vitro Exposure System. | 1-bromopropane; carbon tetrachloride; dichloromethane; trichloroethylene; acrolein; acetaldehyde; formaldehyde;  1,3-butadiene | No | BEAS-2B; pHBEs | ALI | Vapor; Diffusion | 0.021-500 | Exposure: 2 H Collection: 6 H | GC-FID | Cytotoxicity; Metabolism |
| Speit 2008 | Genotoxic effects of formaldehyde in the human lung cell line A549 and in primary human nasal epithelial cells. | formaldehyde | No | A549 | Submerged | Liquid | 0.15 - 15 | 1 H; 4 H; 48 H |  | Genotoxicity; Cell proliferation |
| Speit 2010 | The human lung cell line A549 does not develop adaptive protection against the DNA-damaging action of formaldehyde. | formaldehyde | No | A549 | Submerged | Liquid | 1.5; 3; 6; 9 | 1 H; 4 H; 24 H |  | Genotoxicity; Metabolism |
| Speit 2014 | Investigations on potential co-mutagenic effects of formaldehyde. | formaldehyde | No | A549 | Submerged | Liquid | 0.3; 0.6; 1.5 | 1H |  | Genotoxicity |
| Sthijns 2014 | Adaptation to acrolein through upregulating the protection by glutathione in human bronchial epithelial cells: the materialization of the hormesis concept. | acrolein | No | BEAS-2B | Submerged | Liquid | 0.056 - 11.2 | 0 H; 4 H; 6 H; 8 H; 24 H |  | Cytotoxicity; Other: oxidative stress signaling |
| Su 2023 | Design of a microfluidic lung chip and its application in assessing the toxicity of formaldehyde | formaldehyde | No | A549 | Submerged | Vapor | 1.8 | 0 H |  | Cytotoxicity; ROS generation |
| Tanel 2014 | Acrolein activates cell survival and apoptotic death responses involving the endoplasmic reticulum in A549 lung cells. | acrolein | No | A549 | Submerged | Liquid | 0; 0.84; 1.4; 2.8 | 15 M; 30 M; 2 H |  | Cytotoxicity; Apoptosis; ROS generation; Other: ER stress marker proteins |
| Thompson 2008 | Genome-wide transcriptional responses to acrolein. | acrolein | No | A549 | Submerged | Liquid | 0; 1.4; 2.8; 5.6 | 1; 1.5; 2; 4; 6; 24 H |  | Genotoxicity; Cytotoxicity; Apoptosis; Other: transcriptomics |
| Tian 2017* | Cinnamaldehyde induces cell apoptosis mediated by a novel circular RNA hsa_circ_0043256 in non-small cell lung cancer. | cinnamaldehyde | No | NCI-H460; A549 | Submerged | Liquid | 0; 10; 20; 40 | 24; 48 H |  | Cytotoxicity; Apoptosis; Cell proliferation; Other: miRNA expression |
| Tirumalai 2002 | Acrolein causes transcriptional induction of phase II genes by activation of Nrf2 in human lung type II epithelial (A549) cells. | acrolein | No | A549 | Submerged | Liquid | 28; 42; 56 | 0.5 H; 1H; 3H; 6 H; 12 H; 24 H |  | Cytotoxicity; Other: Oxidative stress signaling |
| Tulen 2022 | Smoking-Associated Exposure of Human Primary Bronchial Epithelial Cells to Aldehydes: Impact on Molecular Mechanisms Controlling Mitochondrial Content and Function. | formaldehyde;  acrolein;  acetaldehyde | Yes | pHBEs | ALI | Vapor | formaldehyde (83), acrolein (59), and acetaldehyde (1162) | 0H; 6 H; 24 H | total carbon analyzer | Genotoxicity; Cytotoxicity; barrier function; Pro-Inflammatory Signaling; Other: oxidative stress signaling pathway, autophagosomal pathway, mitochondrial content and function |
| Valacchi 2005* | Inhibition of NFkappaB activation and IL-8 expression in human bronchial epithelial cells by acrolein. | acrolein | No | pHBEs; HBE1 | Submerged | Liquid | 0.3; 0.6; 1.5 | 6 H; 12 H; 24 H |  | Pro-Inflammatory Signaling |
| Verstraelen 2021 | An In Vitro Air-Liquid Interface Inhalation Platform for Petroleum Substances and Constituents | ethylbenzene | No | A549 | ALI | Vapor | 12,000; 9,500; 7300 | 1 H; 20 H | HS-GC-MS | Cytotoxicity; Pro-Inflammatory Signaling; Other: oxidative stress signaling |
| Vock 1999 | Discrimination between genotoxicity and cytotoxicity for the induction of DNA double-strand breaks in cells treated with aldehydes and diepoxides. | formaldehyde, glutaraldehyde | No | A549 | Submerged | Liquid | formaldehyde (0.003 - 30.03); glutaraldehyde (0.01 - 100) | 8 H, 24 H; 72 H |  | Genotoxicity; Cytotoxicity |
| Wang 2016 | Acrolein preferentially damages nucleolus eliciting ribosomal stress and apoptosis in human cancer cells. | Acrolein | No | A549 | Submerged | Liquid | 1.4 - 14 | 3H; 8H; 24 H |  | Cytotoxicity; Apoptosis |
| Weidinger 2024* | Olfactory receptors impact pathophysiological processes of lung diseases in bronchial epithelial cells. | cinnamaldehyde; brahmanol;  sandalore;  helional | No | A549; pHBEs | Submerged | Liquid | 1.3-105.18 | 20 M; 24 H |  | Apoptosis; barrier function; Pro-Inflammatory Signaling; Cell proliferation; Other: wound healing assay |
| Wisnewski 2002* | Effects of hexamethylene diisocyanate exposure on human airway epithelial cells: In vitro cellular and molecular studies | hexamethylene diisocyanate | No | A549; NCI-H292 | Submerged | Liquid |  | Exposure: 20 M; 24 H Collection: 0 H; 1 H; 2 H; 3 H |  | Cytotoxicity; Other: thioredoxin reductase expression, binding/uptake |
| Wong 2012* | S-phase sensing of DNA-protein crosslinks triggers TopBP1-independent ATR activation and p53-mediated cell death by formaldehyde. | formaldehyde | No | NCI-H460, pHBEs | Submerged | Liquid | 0-9 | 0; 0.5; 1; 2; 3; 6 H |  | Genotoxicity; Apoptosis; Other: cell cycle activity |
| Xiong 2018 | Evaluating Mode of Action of Acrolein Toxicity in an In Vitro Human Airway Tissue Model. | acrolein | No | pHBEs | ALI | Liquid | 7; 14; 28; 42; 56 | 24H |  | Cytotoxicity; Apoptosis; Pro-Inflammatory Signaling; Other: protein carbonylation, oxidative stress signaling, primary cell differentiation |
| Xiong 2021* | In vitro dosimetry analyses for acrolein exposure in normal human lung epithelial cells and human lung cancer cells. | acrolein | No | NHBE; NCI-H292 | Submerged | Liquid | 2.8 | 0; 0.5; 1; 3; 5; 10 M | GC-MS | Cytotoxicity; ROS generation; Other: intracellular protein carbonyl levels |
| Yadav 2013 | Aldose reductase regulates acrolein-induced cytotoxicity in human small airway epithelial cells. | Acrolein | No | SAECs | Submerged | Liquid | 0-1.12 | 0; 2; 12; 24; 48; 72 H 0; 5; 10; 15; 30; 60 M |  | Genotoxicity; Apoptosis; Pro-Inflammatory Signaling; ROS generation; Cell proliferation; Other: cell cycle analysis |
| Yin 2020 | Formation of di-cysteine acrolein adduct decreases cytotoxicity of acrolein by ROS alleviation and apoptosis intervention. | acrolein;  acrolein-di-cys | No | HBE1 | Submerged | Liquid | 0.5 - 10 | 24 H; 48 H; 72 H |  | Cytotoxicity; Apoptosis; ROS generation |
| Zavala 2018 | A new cell culture exposure system for studying the toxicity of volatile chemicals at the air-liquid interface. | acrolein | No | pHBEs | ALI | Vapor | 0.3 | Exposure: 2 H Collection: 0 H; 1 H; 24 H | GC-FID | Cytotoxicity; Pro-Inflammatory Signaling; ROS generation |
| Zhang 2008 | Acrolein induces heme oxygenase-1 through PKC-delta and PI3K in human bronchial epithelial cells. | Acrolein | No | HBE1 | Submerged | Liquid | 0.28 - 2.24 | 12; 24 H |  | Cytotoxicity; Other: oxidative stress signaling |
| Zhang 2009 | Signaling pathways involved in phase II gene induction by alpha, beta-unsaturated aldehydes. | acrolein | No | HBE1 | Submerged | Liquid | 0.28; 0.56; 1.12 | 12 H |  | Pro-Inflammatory Signaling |
| Zhang 2013 | Protective effect of curcumin against formaldehyde-induced genotoxicity in A549 Cell Lines. | formaldehyde |  | A549 | Submerged | Liquid | 3 | 4 H |  | Genotoxicity; Pro-Inflammatory Signaling; Lipid Peroxidation; Other: antioxidant enzyme activity |
| Zhang 2019 | Combined cytotoxicity of co-exposure to aldehyde mixtures on human bronchial epithelial BEAS-2B cells. | formaldehyde; acrolein | Yes | BEAS-2B | Submerged | Liquid | formaldehyde: 0-2.4 acrolein: 0.028-0.42 | 3; 6; 12; 24 H |  | Cytotoxicity; Lipid Peroxidation; ROS generation; Cell proliferation; Other: antioxidant enzyme activity |

**Supplemental Table 3**. General information about each cell line used in at least one study included in this review. N/A = non-applicable because primary cells do not undergo an immortalization method.

**Supplemental Table 4**. A list of all VOC mixtures reported in studies included in this review. Although it is not a VOC, ozone was deemed a valid mixture component in this review.

**Supplemental Figure 1.** A pie chart summarizing the number of studies that reported dose validation for VOC exposures and the breakdown of the reported validation techniques. Approximately a quarter of studies (24.3%) reported dose validation in the experimental design.

**References**

Agarwal, A. R., Yin, F., & Cadenas, E. (2013). Metabolic shift in lung alveolar cell mitochondria following acrolein exposure. Am J Physiol Lung Cell Mol Physiol, 305(10), L764-773. https://doi.org/10.1152/ajplung.00165.2013

Al Zallouha, M., Landkocz, Y., Brunet, J., Cousin, R., Genty, E., Courcot, D., Siffert, S., Shirali, P., & Billet, S. (2017). Usefulness of toxicological validation of VOCs catalytic degradation by air-liquid interface exposure system. Environ Res, 152, 328-335. https://doi.org/10.1016/j.envres.2016.10.027

An, J., Li, F., Qin, Y., Zhang, H., & Ding, S. (2019). Low concentrations of FA exhibits the Hormesis effect by affecting cell division and the Warburg effect. Ecotoxicol Environ Saf, 183, 109576. https://doi.org/10.1016/j.ecoenv.2019.109576

Anderson, S. E., Jackson, L. G., Franko, J., & Wells, J. R. (2010). Evaluation of dicarbonyls generated in a simulated indoor air environment using an in vitro exposure system. Toxicol Sci, 115(2), 453-461. https://doi.org/10.1093/toxsci/kfq067

Anderson, S. E., Khurshid, S. S., Meade, B. J., Lukomska, E., & Wells, J. R. (2013). Toxicological analysis of limonene reaction products using an in vitro exposure system. Toxicol In Vitro, 27(2), 721-730. https://doi.org/10.1016/j.tiv.2012.11.017

Bakand, S., Hayes, A., Winder, C., Khalil, C., & Markovic, B. (2005). In vitro cytotoxicity testing of airborne formaldehyde collected in serum-free culture media. Toxicol Ind Health, 21(7-8), 147-154. https://doi.org/10.1191/0748233705th223oa

Bakand, S., Winder, C., Khalil, C., & Hayes, A. (2006). A novel in vitro exposure technique for toxicity testing of selected volatile organic compounds. J Environ Monit, 8(1), 100-105. https://doi.org/10.1039/b509812b

Baldridge, K. C., Zavala, J., Surratt, J., Sexton, K. G., & Contreras, L. M. (2015). Cellular RNA is chemically modified by exposure to air pollution mixtures. Inhal Toxicol, 27(1), 74-82. https://doi.org/10.3109/08958378.2014.987361

Balharry, D., Sexton, K., & BeruBe, K. A. (2008). An in vitro approach to assess the toxicity of inhaled tobacco smoke components: nicotine, cadmium, formaldehyde and urethane. Toxicology, 244(1), 66-76. https://doi.org/10.1016/j.tox.2007.11.001

Behar, R. Z., Luo, W., McWhirter, K. J., Pankow, J. F., & Talbot, P. (2018). Analytical and toxicological evaluation of flavor chemicals in electronic cigarette refill fluids. Sci Rep, 8(1), 8288. https://doi.org/10.1038/s41598-018-25575-6

Bein, K., Birru, R. L., Wells, H., Larkin, T. P., Cantrell, P. S., Fagerburg, M. V., Zeng, X., & Leikauf, G. D. (2020). Albumin Protects Lung Cells against Acrolein Cytotoxicity and Acrolein-Adducted Albumin Increases Heme Oxygenase 1 Transcripts. Chem Res Toxicol, 33(7), 1969-1979. https://doi.org/10.1021/acs.chemrestox.0c00146

Billet, S., Paget, V., Garcon, G., Heutte, N., Andre, V., Shirali, P., & Sichel, F. (2010). Benzene-induced mutational pattern in the tumour suppressor gene TP53 analysed by use of a functional assay, the functional analysis of separated alleles in yeast, in human lung cells. Arch Toxicol, 84(2), 99-107. https://doi.org/10.1007/s00204-009-0478-z

Binder, S., Rastak, N., Karg, E., Huber, A., Kuhn, E., Dragan, G. C., Monse, C., Breuer, D., Di Bucchianico, S., Delaval, M. N., Oeder, S., Sklorz, M., & Zimmermann, R. (2022). Construction of an In Vitro Air-Liquid Interface Exposure System to Assess the Toxicological Impact of Gas and Particle Phase of Semi-Volatile Organic Compounds. Toxics, 10(12). https://doi.org/10.3390/toxics10120730

Biswal, S., Acquaah-Mensah, G., Datta, K., Wu, X., & Kehrer, J. P. (2002). Inhibition of cell proliferation and AP-1 activity by acrolein in human A549 lung adenocarcinoma cells due to thiol imbalance and covalent modifications. Chem Res Toxicol, 15(2), 180-186. https://doi.org/10.1021/tx015552p

Biswal, S., Maxwell, T., Rangasamy, T., & Kehrer, J. P. (2003). Modulation of benzo[a]pyrene-induced p53 DNA activity by acrolein. Carcinogenesis, 24(8), 1401-1406. https://doi.org/10.1093/carcin/bgg061

Borchers, M. T., Carty, M. P., & Leikauf, G. D. (1999). Regulation of human airway mucins by acrolein and inflammatory mediators. Am J Physiol, 276(4), L549-555. https://doi.org/10.1152/ajplung.1999.276.4.L549

Brostrom, J. M., Ghalali, A., Zheng, H., Hogberg, J., Stenius, U., Littorin, M., Tinnerberg, H., & Broberg, K. (2018). Toluene diisocyanate exposure and autotaxin-lysophosphatidic acid signalling. Toxicol Appl Pharmacol, 355, 43-51. https://doi.org/10.1016/j.taap.2018.06.019

Brostrom, J. M., Ye, Z. W., Axmon, A., Littorin, M., Tinnerberg, H., Lindh, C. H., Zheng, H., Ghalali, A., Stenius, U., Jonsson, B. A., & Hogberg, J. (2015). Toluene diisocyanate: Induction of the autotaxin-lysophosphatidic acid axis and its association with airways symptoms. Toxicol Appl Pharmacol, 287(3), 222-231. https://doi.org/10.1016/j.taap.2015.06.006

Burcham, P. C., Raso, A., & Henry, P. J. (2014). Airborne acrolein induces keratin-8 (Ser-73) hyperphosphorylation and intermediate filament ubiquitination in bronchiolar lung cell monolayers. Toxicology, 319, 44-52. https://doi.org/10.1016/j.tox.2014.02.010

Burcham, P. C., Raso, A., & Kaminskas, L. M. (2012). Chaperone heat shock protein 90 mobilization and hydralazine cytoprotection against acrolein-induced carbonyl stress. Mol Pharmacol, 82(5), 876-886. https://doi.org/10.1124/mol.112.078956

Burcham, P. C., Raso, A., Thompson, C., & Tan, D. (2007). Intermolecular protein cross-linking during acrolein toxicity: efficacy of carbonyl scavengers as inhibitors of heat shock protein-90 cross-linking in A549 cells. Chem Res Toxicol, 20(11), 1629-1637. https://doi.org/10.1021/tx700192e

Burcham, P. C., Raso, A., & Thompson, C. A. (2010a). Intermediate filament carbonylation during acute acrolein toxicity in A549 lung cells: functional consequences, chaperone redistribution, and protection by bisulfite. Antioxid Redox Signal, 12(3), 337-347. https://doi.org/10.1089/ars.2009.2817

Burcham, P. C., Raso, A., & Thompson, C. A. (2010b). Toxicity of smoke extracts towards A549 lung cells: role of acrolein and suppression by carbonyl scavengers. Chem Biol Interact, 183(3), 416-424. https://doi.org/10.1016/j.cbi.2009.12.006

Cheah, N. P., Pennings, J. L., Vermeulen, J. P., van Schooten, F. J., & Opperhuizen, A. (2013). In vitro effects of aldehydes present in tobacco smoke on gene expression in human lung alveolar epithelial cells. Toxicol In Vitro, 27(3), 1072-1081. https://doi.org/10.1016/j.tiv.2013.02.003

Chen, D., Fang, L., Li, H., Tang, M. S., & Jin, C. (2013). Cigarette smoke component acrolein modulates chromatin assembly by inhibiting histone acetylation. J Biol Chem, 288(30), 21678-21687. https://doi.org/10.1074/jbc.M113.476630

Chen, D., Fang, L., Mei, S., Li, H., Xu, X., Des Marais, T. L., Lu, K., Liu, X. S., & Jin, C. (2017). Regulation of Chromatin Assembly and Cell Transformation by Formaldehyde Exposure in Human Cells. Environ Health Perspect, 125(9), 097019. https://doi.org/10.1289/EHP1275

Chen, S. J., Wang, J. L., Chen, J. H., & Huang, R. N. (2002). Possible involvement of glutathione and p53 in trichloroethylene- and perchloroethylene-induced lipid peroxidation and apoptosis in human lung cancer cells. Free Radic Biol Med, 33(4), 464-472. https://doi.org/10.1016/s0891-5849(02)00817-1

Cheng, Y., Kong, D., Ci, M., Guan, Y., Luo, C., Zhang, X., Gao, F., Li, M., & Deng, G. (2023). Oxidative Stress Effects of Multiple Pollutants in an Indoor Environment on Human Bronchial Epithelial Cells. Toxics, 11(3). https://doi.org/10.3390/toxics11030251

Chiung, Y. M., Kao, Y. Y., Chang, W. F., Yao, C. W., & Liu, P. S. (2010). Toluene diisocyanate (TDI) induces calcium elevation and interleukine-4 (IL-4) release - early responses upon TDI stimulation. J Toxicol Sci, 35(2), 197-207. https://doi.org/10.2131/jts.35.197

Cho, Y., Lim, J.-h., Jeong, S.-C., Song, M.-K., & Ryu, J.-C. (2015). Hexanal-induced changes in miRNA-mRNA interactions in A549 human alveolar epithelial cells. Toxicology and Environmental Health Sciences, 7(2), 143-159. https://doi.org/10.1007/s13530-015-0233-1

Cho, Y., Song, M. K., Kim, T. S., & Ryu, J. C. (2018). DNA Methylome Analysis of Saturated Aliphatic Aldehydes in Pulmonary Toxicity. Sci Rep, 8(1), 10497. https://doi.org/10.1038/s41598-018-28813-z

Choi, J. H., Hwang, Y. P., Han, E. H., Kim, H. G., Park, B. H., Lee, H. S., Park, B. K., Lee, Y. C., Chung, Y. C., & Jeong, H. G. (2011). Inhibition of acrolein-stimulated MUC5AC expression by Platycodon grandiflorum root-derived saponin in A549 cells. Food Chem Toxicol, 49(9), 2157-2166. https://doi.org/10.1016/j.fct.2011.05.030

Choi, J. H., Nahm, D. H., Kim, S. H., Kim, Y. S., Suh, C. H., Park, H. S., & Ahn, S. W. (2004). Increased levels of IgG to cytokeratin 19 in sera of patients with toluene diisocyanate-induced asthma. Ann Allergy Asthma Immunol, 93(3), 293-298. https://doi.org/10.1016/S1081-1206(10)61504-9

Cui, Y., Li, H., Wu, S., Zhao, R., Du, D., Ding, Y., Nie, H., & Ji, H. L. (2016). Formaldehyde impairs transepithelial sodium transport. Sci Rep, 6, 35857. https://doi.org/10.1038/srep35857

Deshmukh, H. S., Case, L. M., Wesselkamper, S. C., Borchers, M. T., Martin, L. D., Shertzer, H. G., Nadel, J. A., & Leikauf, G. D. (2005). Metalloproteinases Mediate Mucin 5AC Expression by Epidermal Growth Factor Receptor Activation. American Journal of Respiratory and Critical Care Medicine, 171(4), 305-314. https://doi.org/10.1164/rccm.200408-1003OC

Doyle, M., Sexton, K. G., Jeffries, H., & Jaspers, I. (2007). Atmospheric photochemical transformations enhance 1,3-butadiene-induced inflammatory responses in human epithelial cells: The role of ozone and other photochemical degradation products. Chem Biol Interact, 166(1-3), 163-169. https://doi.org/10.1016/j.cbi.2006.05.016

Du, A., Zhang, S., Miao, J., & Zhao, B. (2004). Safrole oxide induces apoptosis in A549 human lung cancer cells. Exp Lung Res, 30(6), 419-429. https://doi.org/10.1080/01902140490476337

Du, A., Zhao, B., Miao, J., Yin, D., & Zhang, S. (2006). Safrole oxide induces apoptosis by up-regulating Fas and FasL instead of integrin beta4 in A549 human lung cancer cells. Bioorg Med Chem, 14(7), 2438-2445. https://doi.org/10.1016/j.bmc.2005.11.026

Duistermaat, E., de Bruijn, V. M. P., Vermeulen, J. P., Gremmer, E. R., & Vandebriel, R. J. (2025). Towards prospective identification of respiratory sensitizers: effects of piperazine, chloramine-T, and toluene-diisocyanate in an air-liquid interface model comprising human bronchial epithelial cells. Arch Toxicol, 99(6), 2479-2492. https://doi.org/10.1007/s00204-025-04029-9

Dwivedi, A. M., Upadhyay, S., Johanson, G., Ernstgård, L., & Palmberg, L. (2018). Inflammatory effects of acrolein, crotonaldehyde and hexanal vapors on human primary bronchial epithelial cells cultured at air-liquid interface. Toxicol In Vitro, 46, 219-228. https://doi.org/10.1016/j.tiv.2017.09.016

Ebersviller, S., Lichtveld, K., Sexton, K. G., Zavala, J., Lin, Y. H., Jaspers, I., & Jeffries, H. E. (2012). Gaseous VOCs rapidly modify particulate matter and its biological effects - Part 1: Simple VOCs and model PM. Atmos Chem Phys Discuss, 12(2), 5065-5105. https://doi.org/10.5194/acpd-12-5065-2012

Feltens, R., Mogel, I., Roder-Stolinski, C., Simon, J. C., Herberth, G., & Lehmann, I. (2010). Chlorobenzene induces oxidative stress in human lung epithelial cells in vitro. Toxicol Appl Pharmacol, 242(1), 100-108. https://doi.org/10.1016/j.taap.2009.09.020

Feng, Z., Hu, W., Hu, Y., & Tang, M. S. (2006). Acrolein is a major cigarette-related lung cancer agent: Preferential binding at p53 mutational hotspots and inhibition of DNA repair. Proc Natl Acad Sci U S A, 103(42), 15404-15409. https://doi.org/10.1073/pnas.0607031103

Fischader, G., Roder-Stolinski, C., Wichmann, G., Nieber, K., & Lehmann, I. (2008). Release of MCP-1 and IL-8 from lung epithelial cells exposed to volatile organic compounds. Toxicol In Vitro, 22(2), 359-366. https://doi.org/10.1016/j.tiv.2007.09.015

Giuliano, M., Stellavato, A., Cammarota, M., Lamberti, M., Miraglia, N., Sannolo, N., & De Rosa, M. (2009). Effects of low concentrations of benzene on human lung cells in vitro. Toxicol Lett, 188(2), 130-136. https://doi.org/10.1016/j.toxlet.2009.03.018

Gminski, R., Tang, T., & Mersch-Sundermann, V. (2010). Cytotoxicity and genotoxicity in human lung epithelial A549 cells caused by airborne volatile organic compounds emitted from pine wood and oriented strand boards. Toxicol Lett, 196(1), 33-41. https://doi.org/10.1016/j.toxlet.2010.03.015

Gonzalez-Rivera, J. C., Sherman, M. W., Wang, D. S., Chuvalo-Abraham, J. C. L., Hildebrandt Ruiz, L., & Contreras, L. M. (2020). RNA oxidation in chromatin modification and DNA-damage response following exposure to formaldehyde. Sci Rep, 10(1), 16545. https://doi.org/10.1038/s41598-020-73376-7

Gostner, J. M., Zeisler, J., Alam, M. T., Gruber, P., Fuchs, D., Becker, K., Neubert, K., Kleinhappl, M., Martini, S., & Uberall, F. (2016). Cellular reactions to long-term volatile organic compound (VOC) exposures. Sci Rep, 6, 37842. https://doi.org/10.1038/srep37842

Grafstrom, R. C., Dypbukt, J. M., Willey, J. C., Sundqvist, K., Edman, C., Atzori, L., & Harris, C. C. (1988). Pathobiological effects of acrolein in cultured human bronchial epithelial cells. Cancer Res, 48(7), 1717-1721. https://www.ncbi.nlm.nih.gov/pubmed/3349453

Han, D. H., Shin, M. K., Oh, J. W., Lee, J., Sung, J. S., & Kim, M. (2023). Chronic Exposure to TDI Induces Cell Migration and Invasion via TGF-beta1 Signal Transduction. Int J Mol Sci, 24(7). https://doi.org/10.3390/ijms24076157

Horton, N. D., Biswal, S. S., Corrigan, L. L., Bratta, J., & Kehrer, J. P. (1999). Acrolein causes inhibitor kappaB-independent decreases in nuclear factor kappaB activation in human lung adenocarcinoma (A549) cells. J Biol Chem, 274(14), 9200-9206. https://doi.org/10.1074/jbc.274.14.9200

Horton, N. D., Mamiya, B. M., & Kehrer, J. P. (1997). Relationships between cell density, glutathione and proliferation of A549 human lung adenocarcinoma cells treated with acrolein. Toxicology, 122(1), 111-122. https://doi.org/https://doi.org/10.1016/S0300-483X(97)00086-3

Jiang, K., Yin, Z., Zhou, P., Guo, H., Huang, C., Zhang, G., Hu, W., Ou, S., & Ou, J. (2020). The scavenging capacity of gamma-aminobutyric acid for acrolein and the cytotoxicity of the formed adduct. Food Funct, 11(9), 7736-7747. https://doi.org/10.1039/c9fo02518a

Johanson, G., Dwivedi, A. M., Ernstgård, L., Palmberg, L., Ganguly, K., Chen, L. C., Galdanes, K., Gordon, T., & Upadhyay, S. (2020). Analysis of Acrolein Exposure Induced Pulmonary Response in Seven Inbred Mouse Strains and Human Primary Bronchial Epithelial Cells Cultured at Air-Liquid Interface. Biomed Res Int, 2020, 3259723. https://doi.org/10.1155/2020/3259723

Jude, J., Koziol-White, C., Scala, J., Yoo, E., Jester, W., Maute, C., Dalton, P., & Panettieri, R., Jr. (2016). Formaldehyde Induces Rho-Associated Kinase Activity to Evoke Airway Hyperresponsiveness. Am J Respir Cell Mol Biol, 55(4), 542-553. https://doi.org/10.1165/rcmb.2015-0254OC

Kastner, P. E., Casset, A., & Pons, F. (2011). Formaldehyde interferes with airway epithelium integrity and functions in a dose- and time-dependent manner. Toxicol Lett, 200(1-2), 109-116. https://doi.org/10.1016/j.toxlet.2010.11.003

Kastner, P. E., Le Calve, S., Zheng, W., Casset, A., & Pons, F. (2013). A dynamic system for single and repeated exposure of airway epithelial cells to gaseous pollutants. Toxicol In Vitro, 27(2), 632-640. https://doi.org/10.1016/j.tiv.2012.11.011

Kim, J. H., Jang, Y. S., Jang, S. H., Jung, K. S., Kim, S. H., Ye, Y. M., & Park, H. S. (2017). Toluene diisocyanate exposure induces airway inflammation of bronchial epithelial cells via the activation of transient receptor potential melastatin 8. Exp Mol Med, 49(3), e299. https://doi.org/10.1038/emm.2016.161

Kim, S., Kim, M., & Sung, J. S. (2022). Exposure of Toluene Diisocyanate Induces DUSP6 and p53 through Activation of TRPA1 Receptor. Int J Mol Sci, 23(1). https://doi.org/10.3390/ijms23010517

Kim, S. H., Choi, G. S., Ye, Y. M., Jou, I., Park, H. S., & Park, S. M. (2010). Toluene diisocyanate (TDI) regulates haem oxygenase-1/ferritin expression: implications for toluene diisocyanate-induced asthma. Clin Exp Immunol, 160(3), 489-497. https://doi.org/10.1111/j.1365-2249.2010.04118.x

Kim, Y.-R., Kim, I.-K., Lee, S. H., Lee, E.-J., Pyo, M. J., Lee, H. R., Youn, J. P., Oh, M., Hwang, S. Y., & Kim, M.-K. (2012). Gene expression profiles of human lung epithelial cells exposed to toluene. Toxicology and Environmental Health Sciences, 4(4), 269-276. https://doi.org/10.1007/s13530-012-0146-1

Komori, K., Murai, K., Miyajima, S., Fujii, T., Mohri, S., Ono, Y., & Sakai, Y. (2008). Development of an in vitro batch-type closed gas exposure device with an alveolar epithelial cell line, A549, for toxicity evaluations of gaseous compounds. Anal Sci, 24(8), 957-962. https://doi.org/10.2116/analsci.24.957

Lange, R. W., Lantz, R. C., Stolz, D. B., Watkins, S. C., Sundareshan, P., Lemus, R., & Karol, M. H. (1999). Toluene diisocyanate colocalizes with tubulin on cilia of differentiated human airway epithelial cells. Toxicol Sci, 50(1), 64-71. https://doi.org/10.1093/toxsci/50.1.64

Lantz, R. C., Lemus, R., Lange, R. W., & Karol, M. H. (2001). Rapid reduction of intracellular glutathione in human bronchial epithelial cells exposed to occupational levels of toluene diisocyanate. Toxicol Sci, 60(2), 348-355. https://doi.org/10.1093/toxsci/60.2.348

Le, Y., Ren, B., Muskhelishvili, L., Davis, K., Wang, Y., Gwinn, W., Rua, D., Heflich, R. H., & Cao, X. (2025). Characterizing the Pulmonary Toxicity and Potential Mutagenicity of Formaldehyde Fumes in a Human Bronchial Epithelial Tissue Model. Environ Mol Mutagen, 66(1-2), 6-21. https://doi.org/10.1002/em.70000

Lecureur, V., Arzel, M., Ameziane, S., Houlbert, N., Le Vee, M., Jouneau, S., & Fardel, O. (2012). MAPK- and PKC/CREB-dependent induction of interleukin-11 by the environmental contaminant formaldehyde in human bronchial epithelial cells. Toxicology, 292(1), 13-22. https://doi.org/10.1016/j.tox.2011.11.011

Lee, H. S., Yoon, J.-S., Song, M., Shin, C.-Y., Chung, H. S., & Ryu, J.-C. (2012). Gene expression profiling of low dose exposure of saturated aliphatic aldehydes in A549 human alveolar epithelial cells. Toxicology and Environmental Health Sciences, 4(4), 211-217. https://doi.org/10.1007/s13530-012-0140-7

Lehmann, I., Roder-Stolinski, C., Nieber, K., & Fischader, G. (2008). In vitro models for the assessment of inflammatory and immuno-modulatory effects of the volatile organic compound chlorobenzene. Exp Toxicol Pathol, 60(2-3), 185-193. https://doi.org/10.1016/j.etp.2008.01.009

Lim, S. K., Choi, H., Park, M. J., Kim, D. I., Kim, J. C., Kim, G. Y., Jeong, S. Y., Rodionov, R. N., Han, H. J., Yoon, K. C., & Park, S. H. (2013). The ER stress-mediated decrease in DDAH1 expression is involved in formaldehyde-induced apoptosis in lung epithelial cells. Food Chem Toxicol, 62, 763-769. https://doi.org/10.1016/j.fct.2013.10.014

Lim, S. K., Kim, J. C., Moon, C. J., Kim, G. Y., Han, H. J., & Park, S. H. (2010). Formaldehyde induces apoptosis through decreased Prx 2 via p38 MAPK in lung epithelial cells. Toxicology, 271(3), 100-106. https://doi.org/10.1016/j.tox.2010.03.011

Lim, S. K., Yoo, J., Kim, H., Lim, Y. M., Kim, W., Shim, I., Kim, H. R., Kim, P., & Eom, I. C. (2021). Prediction of acute inhalation toxicity using cytotoxicity data from human lung epithelial cell lines. J Appl Toxicol, 41(7), 1038-1049. https://doi.org/10.1002/jat.4090

Lin, Y. H., Sexton, K. G., Jaspers, I., Li, Y. R., Surratt, J. D., & Vizuete, W. (2014). Application of chemical vapor generation systems to deliver constant gas concentrations for in vitro exposure to volatile organic compounds. Environ Sci Process Impacts, 16(12), 2703-2710. https://doi.org/10.1039/c4em00465e

Liu, D., Cheng, Y., Tang, Z., Mei, X., Cao, X., & Liu, J. (2022). Toxicity mechanism of acrolein on DNA damage and apoptosis in BEAS-2B cells: Insights from cell biology and molecular docking analyses. Toxicology, 466, 153083. https://doi.org/10.1016/j.tox.2021.153083

Liu, F. F., Escher, B. I., Were, S., Duffy, L., & Ng, J. C. (2014). Mixture effects of benzene, toluene, ethylbenzene, and xylenes (BTEX) on lung carcinoma cells via a hanging drop air exposure system. Chem Res Toxicol, 27(6), 952-959. https://doi.org/10.1021/tx5000552

Liu, F. F., Peng, C., Escher, B. I., Fantino, E., Giles, C., Were, S., Duffy, L., & Ng, J. C. (2013). Hanging drop: an in vitro air toxic exposure model using human lung cells in 2D and 3D structures. J Hazard Mater, 261, 701-710. https://doi.org/10.1016/j.jhazmat.2013.01.027

Liu, F. F., Peng, C., & Ng, J. C. (2015). BTEX in vitro exposure tool using human lung cells: trips and gains. Chemosphere, 128, 321-326. https://doi.org/10.1016/j.chemosphere.2015.01.058

Liu, Q., Yang, L., Gong, C., Tao, G., Huang, H., Liu, J., Zhang, H., Wu, D., Xia, B., Hu, G., Wang, K., & Zhuang, Z. (2011). Effects of long-term low-dose formaldehyde exposure on global genomic hypomethylation in 16HBE cells. Toxicol Lett, 205(3), 235-240. https://doi.org/10.1016/j.toxlet.2011.05.1039

Liu, X. Y., Yang, Z. H., Pan, X. J., Zhu, M. X., & Xie, J. P. (2010). Gene expression profile and cytotoxicity of human bronchial epithelial cells exposed to crotonaldehyde. Toxicol Lett, 197(2), 113-122. https://doi.org/10.1016/j.toxlet.2010.05.005

Lv, Y., Gan, S., Chen, Z., Luo, T., Yang, C., Fu, L., Lin, L., Yao, L., & Tang, H. (2024). GLUT1 mediates bronchial epithelial E-cadherin disruption in TDI-induced steroid-insensitive asthma. J Asthma, 61(11), 1571-1580. https://doi.org/10.1080/02770903.2024.2368199

Mascelloni, M., Delgado-Saborit, J. M., Hodges, N. J., & Harrison, R. M. (2015). Study of gaseous benzene effects upon A549 lung epithelial cells using a novel exposure system. Toxicol Lett, 237(1), 38-45. https://doi.org/10.1016/j.toxlet.2015.05.015

Mattoli, S., Colotta, F., Fincato, G., Mezzetti, M., Mantovani, A., Patalano, F., & Fasoli, A. (1991). Time course of IL1 and IL6 synthesis and release in human bronchial epithelial cell cultures exposed to toluene diisocyanate. J Cell Physiol, 149(2), 260-268. https://doi.org/10.1002/jcp.1041490212

Meausoone, C., El Khawaja, R., Tremolet, G., Siffert, S., Cousin, R., Cazier, F., Billet, S., Courcot, D., & Landkocz, Y. (2019). In vitro toxicological evaluation of emissions from catalytic oxidation removal of industrial VOCs by air/liquid interface (ALI) exposure system in repeated mode. Toxicol In Vitro, 58, 110-117. https://doi.org/10.1016/j.tiv.2019.03.030

Meausoone, C., Landkocz, Y., Cazier, F., Seigneur, M., Courcot, D., & Billet, S. (2021). Toxicological responses of BEAS-2B cells to repeated exposures to benzene, toluene, m-xylene, and mesitylene using air-liquid interface method. J Appl Toxicol, 41(8), 1262-1274. https://doi.org/10.1002/jat.4113

Mogel, I., Baumann, S., Bohme, A., Kohajda, T., von Bergen, M., Simon, J. C., & Lehmann, I. (2011). The aromatic volatile organic compounds toluene, benzene and styrene induce COX-2 and prostaglandins in human lung epithelial cells via oxidative stress and p38 MAPK activation. Toxicology, 289(1), 28-37. https://doi.org/10.1016/j.tox.2011.07.006

Morbt, N., Mogel, I., Kalkhof, S., Feltens, R., Roder-Stolinski, C., Zheng, J., Vogt, C., Lehmann, I., & von Bergen, M. (2009). Proteome changes in human bronchoalveolar cells following styrene exposure indicate involvement of oxidative stress in the molecular-response mechanism. Proteomics, 9(21), 4920-4933. https://doi.org/10.1002/pmic.200800836

Mörbt, N., Tomm, J., Feltens, R., Mögel, I., Kalkhof, S., Murugesan, K., Wirth, H., Vogt, C., Binder, H., Lehmann, I., & von Bergen, M. (2011). Chlorinated benzenes cause concomitantly oxidative stress and induction of apoptotic markers in lung epithelial cells (A549) at nonacute toxic concentrations. J Proteome Res, 10(2), 363-378. https://doi.org/10.1021/pr1005718

Moretto, N., Bertolini, S., Iadicicco, C., Marchini, G., Kaur, M., Volpi, G., Patacchini, R., Singh, D., & Facchinetti, F. (2012). Cigarette smoke and its component acrolein augment IL-8/CXCL8 mRNA stability via p38 MAPK/MK2 signaling in human pulmonary cells. Am J Physiol Lung Cell Mol Physiol, 303(10), L929-938. https://doi.org/10.1152/ajplung.00046.2012

Murugesan, K., Baumann, S., Wissenbach, D. K., Kliemt, S., Kalkhof, S., Otto, W., Mogel, I., Kohajda, T., von Bergen, M., & Tomm, J. M. (2013). Subtoxic and toxic concentrations of benzene and toluene induce Nrf2-mediated antioxidative stress response and affect the central carbon metabolism in lung epithelial cells A549. Proteomics, 13(21), 3211-3221. https://doi.org/10.1002/pmic.201300126

Myers, C. R., & Myers, J. M. (2009). The effects of acrolein on peroxiredoxins, thioredoxins, and thioredoxin reductase in human bronchial epithelial cells. Toxicology, 257(1-2), 95-104. https://doi.org/10.1016/j.tox.2008.12.013

Najera-Martinez, M., Garcia-Latorre, E. A., Reyes-Maldonado, E., Dominguez-Lopez, M. L., & Vega-Lopez, A. (2012). Halomethane-induced cytotoxicity and cell proliferation in human lung MRC-5 fibroblasts and NL20-TA epithelial cells. Inhal Toxicol, 24(11), 762-773. https://doi.org/10.3109/08958378.2012.716871

Nardini, M., Finkelstein, E. I., Reddy, S., Valacchi, G., Traber, M., Cross, C. E., & van der Vliet, A. (2002). Acrolein-induced cytotoxicity in cultured human bronchial epithelial cells. Modulation by alpha-tocopherol and ascorbic acid. Toxicology, 170(3), 173-185. https://doi.org/10.1016/s0300-483x(01)00540-6

Neuss, S., & Speit, G. (2008). Further characterization of the genotoxicity of formaldehyde in vitro by the sister chromatid exchange test and co-cultivation experiments. Mutagenesis, 23(5), 355-357. https://doi.org/10.1093/mutage/gen025

Ortega-Atienza, S., Green, S. E., & Zhitkovich, A. (2015). Proteasome activity is important for replication recovery, CHK1 phosphorylation and prevention of G2 arrest after low-dose formaldehyde. Toxicology and Applied Pharmacology, 286(2), 135–41. 10.1016/j.taap.2015.03.018

Pariselli, F., Sacco, M. G., Ponti, J., & Rembges, D. (2009a). Effects of toluene and benzene air mixtures on human lung cells (A549). Experimental and Toxicologic Pathology : Official Journal of the Gesellschaft Fur Toxikologische Pathologie, 61(4), 381–6. 10.1016/j.etp.2008.10.004

Pariselli, F., Sacco, M. G., & Rembges, D. (2009b). An optimized method for in vitro exposure of human derived lung cells to volatile chemicals. Experimental and Toxicologic Pathology : Official Journal of the Gesellschaft Fur Toxikologische Pathologie, 61(1), 33–9. 10.1016/j.etp.2008.05.008

Park, J., & Baek, S. H. (2020). Combination Therapy with Cinnamaldehyde and Hyperthermia Induces Apoptosis of A549 Non-Small Cell Lung Carcinoma Cells via Regulation of Reactive Oxygen Species and Mitogen-Activated Protein Kinase Family. International Journal of Molecular Sciences, 21(17)10.3390/ijms21176229

Persoz, C., Achard, S., Momas, I., & Seta, N. (2012). Inflammatory response modulation of airway epithelial cells exposed to formaldehyde. Toxicology Letters, 211(2), 159–163. 10.1016/j.toxlet.2012.03.799

Persoz, C., Achard, S., Leleu, C., Momas, I., & Seta, N. (2010). An in vitro model to evaluate the inflammatory response after gaseous formaldehyde exposure of lung epithelial cells. Toxicology Letters, 195(2-3), 99–105. 10.1016/j.toxlet.2010.03.003

Persoz, C., Leleu, C., Achard, S., Fasseu, M., Menotti, J., Meneceur, P., Momas, I., Derouin, F., & Seta, N. (2011). Sequential air-liquid exposure of human respiratory cells to chemical and biological pollutants. Toxicology Letters, 207(1), 53–9. 10.1016/j.toxlet.2011.07.028

Pokharel, Y. R., Yoon, S. Y., Kim, S. K., Li, J., & Kang, K. W. (2008). Inhibition of acrolein-stimulated MUC5AC production by fucoidan in human bronchial epithelial cells. Die Pharmazie, 63(10), 757–9. https://ovidsp.ovid.com/ovidweb.cgi?T=JS&CSC=Y&NEWS=N&PAGE=fulltext&D=med7&AN=18972840http://linkresolver.tamu.edu:9003/tamu?sid=OVID:medline&id=pmid:18972840&id=doi:&issn=0031-7144&isbn=&volume=63&issue=10&spage=757&pages=757-9&date=2008&title=Pharmazie&

Rager J.E., Smeester L., Jaspers I., Sexton K.G., & Fry R.C. (2010). Formaldehyde exposure alters microrna expression profiles in human lung cells. Environmental and Molecular Mutagenesis, 51(7), 735. 10.1002/em.20625

Rager, J. E., Smeester, L., Jaspers, I., Sexton, K. G., & Fry, R. C. (2011). Epigenetic changes induced by air toxics: formaldehyde exposure alters miRNA expression profiles in human lung cells. Environmental Health Perspectives, 119(4), 494–500. 10.1289/ehp.1002614

Randall, M. J., Hristova, M., & van der Vliet, A. (2013). Protein alkylation by the alpha,beta-unsaturated aldehyde acrolein. A reversible mechanism of electrophile signaling? FEBS Letters, 587(23), 3808–14. 10.1016/j.febslet.2013.10.006

Randall, M. J., Spiess, P. C., Hristova, M., Hondal, R. J., & van der Vliet, A. (2013). Acrolein-induced activation of mitogen-activated protein kinase signaling is mediated by alkylation of thioredoxin reductase and thioredoxin 1. Redox Biology, 1(101605639), 265–75. 10.1016/j.redox.2013.02.001

Roder-Stolinski, C., Fischader, G., Oostingh, G. J., Eder, K., Duschl, A., & Lehmann, I. (2008). Chlorobenzene induces the NF-kappa B and p38 MAP kinase pathways in lung epithelial cells. Inhalation Toxicology, 20(9), 813–20. 10.1080/08958370802020810

Roder-Stolinski, C., Fischader, G., Oostingh, G. J., Feltens, R., Kohse, F., von Bergen, M., Morbt, N., Eder, K., Duschl, A., & Lehmann, I. (2008). Styrene induces an inflammatory response in human lung epithelial cells via oxidative stress and NF-kappaB activation. Toxicology and Applied Pharmacology, 231(2), 241–7. 10.1016/j.taap.2008.04.010

Roy, J., Pallepati, P., Bettaieb, A., & Averill-Bates, D. (2010). Acrolein induces apoptosis through the death receptor pathway in A549 lung cells: role of p53. Canadian Journal of Physiology and Pharmacology, 88(3), 353–68. 10.1139/Y09-134

Roy, J., Pallepati, P., Bettaieb, A., Tanel, A., & Averill-Bates, D. (2009). Acrolein induces a cellular stress response and triggers mitochondrial apoptosis in A549 cells. Chemico-Biological Interactions, 181(2), 154–67. 10.1016/j.cbi.2009.07.001

Sakolish, C., Georgescu, A., Huh, D. D., & Rusyn, I. (2022). A Model of Human Small Airway on a Chip for Studies of Subacute Effects of Inhalation Toxicants. Toxicological Sciences, 187(2), 267–278. 10.1093/toxsci/kfac036

Saladino, A. J., Willey, J. C., Lechner, J. F., Grafstrom, R. C., LaVeck, M., & Harris, C. C. (1985). Effects of formaldehyde, acetaldehyde, benzoyl peroxide, and hydrogen peroxide on cultured normal human bronchial epithelial cells. Cancer Research, 45(6), 2522–6. https://ovidsp.ovid.com/ovidweb.cgi?T=JS&CSC=Y&NEWS=N&PAGE=fulltext&D=med2&AN=3986791http://linkresolver.tamu.edu:9003/tamu?sid=OVID:medline&id=pmid:3986791&id=doi:&issn=0008-5472&isbn=&volume=45&issue=6&spage=2522&pages=2522-6&date=1985&title=Cancer+Rese

Sarkar, P. (2019). Response of DNA damage genes in acrolein-treated lung adenocarcinoma cells. Molecular and Cellular Biochemistry, 450(1-2), 187–198. 10.1007/s11010-018-3385-x

Sauer U.G., Vogel S., Hess A., Kolle S.N., Ma-Hock L., van Ravenzwaay B., & Landsiedel R. (2013). In vivo-in vitro comparison of acute respiratory tract toxicity using human 3D airway epithelial models and human A549 and murine 3T3 monolayer cell systems. Toxicology in Vitro, 27(1), 174EP–190. 10.1016/j.tiv.2012.10.007

Sayyed, K., Nour-ElDine, W., Rufka, A., Mehanna, S., Khnayzer, R. S., Abi-Gerges, A., & Khalil, C. (2022). Acute cytotoxicity, genotoxicity, and apoptosis induced by petroleum VOC emissions in A549 cell line. Toxicology in Vitro: An International Journal Published in Association with BIBRA, 83(8712158), 105409. 10.1016/j.tiv.2022.105409

Song, M. K., Choi, H. S., Lee, H. S., Kim, Y. J., Park, Y. K., & Ryu, J. C. (2014). Analysis of microRNA and mRNA expression profiles highlights alterations in modulation of the MAPK pathway under octanal exposure. Environmental Toxicology and Pharmacology, 37(1), 84–94. 10.1016/j.etap.2013.11.005

Song, M. K., Lee, H. S., & Ryu, J. C. (2015). Integrated analysis of microRNA and mRNA expression profiles highlights aldehyde-induced inflammatory responses in cells relevant for lung toxicity. Toxicology, 334, 111–121. 10.1016/j.tox.2015.06.007

Song, M., Choi, H., Lee, H., & Ryu, J. (2014). Transcriptome profile analysis of saturated aliphatic aldehydes reveals carbon number-specific molecules involved in pulmonary toxicity. Chemical Research in Toxicology, 27(8), 1362–70. 10.1021/tx500171r

Speit, G., Linsenmeyer, R., Duong, G., & Bausinger, J. (2014). Investigations on potential co-mutagenic effects of formaldehyde. Mutation Research, 760, 48–56. 10.1016/j.mrfmmm.2013.11.007

Speit, G., Neuss, S., & Schmid, O. (2010). The human lung cell line A549 does not develop adaptive protection against the DNA-damaging action of formaldehyde. Environmental and Molecular Mutagenesis, 51(2), 130–7. 10.1002/em.20524

Speit, G., Schmid, O., Neuss, S., & Schutz, P. (2008). Genotoxic effects of formaldehyde in the human lung cell line A549 and in primary human nasal epithelial cells. Environmental and Molecular Mutagenesis, 49(4), 300–7. 10.1002/em.20386

Sthijns, M. M. J. P. E., Randall, M. J., Bast, A., & Haenen, G. R. M. M. (2014). Adaptation to acrolein through upregulating the protection by glutathione in human bronchial epithelial cells: the materialization of the hormesis concept. Biochemical and Biophysical Research Communications, 446(4), 1029–34. 10.1016/j.bbrc.2014.03.081

Su M., Li X., Li Z., Hua C., Shang P., Zhao J., Liu K., & Xie F. (2023). Design of a microfluidic lung chip and its application in assessing the toxicity of formaldehyde. Toxicology Mechanisms and Methods, 33(5), 427EP–436. 10.1080/15376516.2022.2159903

Tanel, A., Pallepati, P., Bettaieb, A., Morin, P., & Averill-Bates, D. (2014). Acrolein activates cell survival and apoptotic death responses involving the endoplasmic reticulum in A549 lung cells. Biochimica Et Biophysica Acta, 1843(5), 827–35. 10.1016/j.bbamcr.2013.12.012

Thompson, C. A., & Burcham, P. C. (2008). Genome-wide transcriptional responses to acrolein. Chemical Research in Toxicology, 21(12), 2245–56. 10.1021/tx8001934

Tian, F., Yu, C. T., Ye, W. D., & Wang, Q. (2017). Cinnamaldehyde induces cell apoptosis mediated by a novel circular RNA hsa_circ_0043256 in non-small cell lung cancer. Biochemical and Biophysical Research Communications, 493(3), 1260–1266. 10.1016/j.bbrc.2017.09.136

Tirumalai, R., Rajesh Kumar, T., Mai, K. H., & Biswal, S. (2002). Acrolein causes transcriptional induction of phase II genes by activation of Nrf2 in human lung type II epithelial (A549) cells. Toxicology Letters, 132(1), 27–36. 10.1016/s0378-4274(02)00055-3

Tulen, C. B. M., Duistermaat, E., Cremers, J. W. J. M., Klerx, W. N. M., Fokkens, P. H. B., Weibolt, N., Kloosterboer, N., Dentener, M. A., Gremmer, E. R., Jessen, P. J. J., Koene, E. J. C., Maas, L., Opperhuizen, A., van Schooten, F., Staal, Y. C. M., & Remels, A. H. V. (2022). Smoking-Associated Exposure of Human Primary Bronchial Epithelial Cells to Aldehydes: Impact on Molecular Mechanisms Controlling Mitochondrial Content and Function. Cells, 11(21)10.3390/cells11213481

Valacchi, G., Pagnin, E., Phung, A., Nardini, M., Schock, B. C., Cross, C. E., & van der Vliet, A. (2005). Inhibition of NFkappaB activation and IL-8 expression in human bronchial epithelial cells by acrolein. Antioxidants & Redox Signaling, 7(1-2), 25–31. 10.1089/ars.2005.7.25

Verstraelen, S., Jacobs, A., Van Laer, J., Hollanders, K., Van Deun, M., Bertels, D., Brabers, R., Witters, H., Remy, S., Geerts, L., Deferme, L., & Frijns, E. (2021). An In Vitro Air-Liquid Interface Inhalation Platform for Petroleum Substances and Constituents. Altex-Alternatives to Animal Experimentation, 38(4), 550–564. 10.14573/altex.2010211

Vock, E. H., Lutz, W. K., Ilinskaya, O., & Vamvakas, S. (1999). Discrimination between genotoxicity and cytotoxicity for the induction of DNA double-strand breaks in cells treated with aldehydes and diepoxides. Mutation Research, 441(1), 85–93. 10.1016/s1383-5718(99)00038-8

Wang, H., Chen, T., Weng, C., Yang, C., & Tang, M. (2016). Acrolein preferentially damages nucleolus eliciting ribosomal stress and apoptosis in human cancer cells. Oncotarget, 7(49), 80450–80464. 10.18632/oncotarget.12608

Weidinger, D., Jacobsen, J., Alisch, D., Uebner, H., Heinen, N., Greune, L., Westhoven, S., Jamal Jameel, K., Kronsbein, J., Pfaender, S., Taube, C., Reuter, S., Peters, M., Hatt, H., & Knobloch, J. (2024). Olfactory receptors impact pathophysiological processes of lung diseases in bronchial epithelial cells. European Journal of Cell Biology, 103(2), 151408. 10.1016/j.ejcb.2024.151408

Wisnewski, A. V., Liu, O., Miller, J. J., Magoski, N., & Redlich, C. A. (2002). Effects of hexamethylene diisocyanate exposure on human airway epithelial cells:: In vitro cellular and molecular studies. Environmental Health Perspectives, 110(9), 901–906. 10.1289/ehp.02110901

Wong, V. C., Cash, H. L., Morse, J. L., Lu, S., & Zhitkovich, A. (2012). S-phase sensing of DNA-protein crosslinks triggers TopBP1-independent ATR activation and p53-mediated cell death by formaldehyde. Cell Cycle (Georgetown, Tex.), 11(13), 2526–37. 10.4161/cc.20905

Xiong, R., Wu, Q., Bryant, M., Rosenfeldt, H., Healy, S., & Cao, X. (2021). In vitro dosimetry analyses for acrolein exposure in normal human lung epithelial cells and human lung cancer cells. Environmental Toxicology and Pharmacology, 83(9612020), 103576. 10.1016/j.etap.2020.103576

Xiong, R., Wu, Q., Muskhelishvili, L., Davis, K., Shemansky, J. M., Bryant, M., Rosenfeldt, H., Healy, S. M., & Cao, X. (2018). Evaluating Mode of Action of Acrolein Toxicity in an In Vitro Human Airway Tissue Model. Toxicological Sciences: An Official Journal of the Society of Toxicology, 166(2), 451–464. 10.1093/toxsci/kfy226

Yadav, U. C. S., Ramana, K. V., & Srivastava, S. K. (2013). Aldose reductase regulates acrolein-induced cytotoxicity in human small airway epithelial cells. Free Radical Biology & Medicine, 65(8709159), 15–25. 10.1016/j.freeradbiomed.2013.06.008

Yin, Z., Jiang, K., Shi, L., Fei, J., Zheng, J., Ou, S., & Ou, J. (2020). Formation of di-cysteine acrolein adduct decreases cytotoxicity of acrolein by ROS alleviation and apoptosis intervention. Journal of Hazardous Materials, 387, 121686. 10.1016/j.jhazmat.2019.121686

Zavala, J., Ledbetter, A. D., Morgan, D. S., Dailey, L. A., Puckett, E., McCullough, S. D., & Higuchi, M. (2018). A new cell culture exposure system for studying the toxicity of volatile chemicals at the air-liquid interface. Inhalation Toxicology, 30(4-5), 169–177. 10.1080/08958378.2018.14839

Zhang, B., Shi, Y., Chen, X., Dai, J., Jiang, Z., Li, N., & Zhang, Z. (2013). Protective effect of curcumin against formaldehyde-induced genotoxicity in A549 Cell Lines. Journal of Applied Toxicology: JAT, 33(12), 1468–73. 10.1002/jat.2814

Zhang, H., & Forman, H. J. (2008). Acrolein induces heme oxygenase-1 through PKC-delta and PI3K in human bronchial epithelial cells. American Journal of Respiratory Cell and Molecular Biology, 38(4), 483–90. 10.1165/rcmb.2007-0260OC

Zhang, H., & Forman, H. J. (2009). Signaling pathways involved in phase II gene induction by alpha, beta-unsaturated aldehydes. Toxicology and Industrial Health, 25(4-5), 269–78. 10.1177/0748233709102209

Zhang, S., Zhang, J., Chen, H., Wang, A., Liu, Y., Hou, H., & Hu, Q. (2019). Combined cytotoxicity of co-exposure to aldehyde mixtures on human bronchial epithelial BEAS-2B cells. Environmental Pollution (Barking, Essex : 1987), 250, 650–661. 10.1016/j.envpol.2019.03.118
